# Acinar-ductal cell rearrangement drives branching morphogenesis of the murine pancreas in an IGF/PI3K-dependent manner

**DOI:** 10.1016/j.devcel.2023.12.011

**Published:** 2024-02-05

**Authors:** Jean-Francois Darrigrand, Anna Salowka, Alejo Torres-Cano, Rafael Tapia-Rojo, Tong Zhu, Sergi Garcia-Manyes, Francesca M. Spagnoli

**Affiliations:** 1 Centre for Gene Therapy and Regenerative Medicine, King’s College London, London, Great Maze Pond, SE1 9RT London, UK; 2 Department of Physics, London Centre for Nanotechnology, King’s College London, London, UK; 3 Department of Physics, Randall Centre for Cell and Molecular Biophysics, Centre for the Physical Science of Life and London Centre for Nanotechnology, King’s College London, London, UK; 4 Single-Molecule Mechanobiology Laboratory, The Francis Crick Institute, London, UK

**Keywords:** branching morphogenesis, tissue fluidity, IGF/PI3K, pancreas, acinar-ductal architecture, ECM remodeling, pancreatic ducts, pancreatic acinar cells

## Abstract

During organ formation, progenitor cells need to acquire different cell identities and organize themselves into distinct structural units. How these processes are coordinated and how tissue architecture(s) is preserved despite the dramatic cell rearrangements occurring in developing organs remain unclear. Here, we identified cellular rearrangements between acinar and ductal progenitors as a mechanism to drive branching morphogenesis in the pancreas while preserving the integrity of the acinar-ductal functional unit. Using *ex vivo* and *in vivo* mouse models, we found that pancreatic ductal cells form clefts by protruding and pulling on the acinar basement membrane, which leads to acini splitting. Newly formed acini remain connected to the bifurcated branches generated by ductal cell rearrangement. Insulin growth factor (IGF)/phosphatidylinositol 3-kinase (PI3K) pathway finely regulates this process by controlling pancreatic ductal tissue fluidity, with a simultaneous impact on branching and cell fate acquisition. Together, our results explain how acinar structure multiplication and branch bifurcation are synchronized during pancreas organogenesis.

## Highlights

• Rearrangements between acinar and ductal progenitors drive branching in the pancreas
• Pancreatic ductal cells form clefts by protruding and pulling on the acinar BM
• IGF/PI3K pathway regulates cellular rearrangements between acinar and ductal cells

## Graphical abstract

**Figure:**
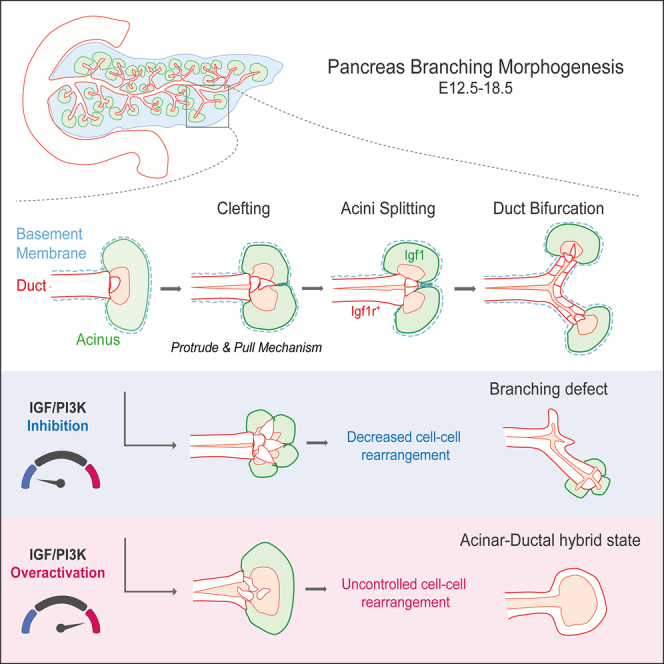

## Teaser

Darrigrand et al. identified that cellular rearrangements between acinar and ductal progenitors drive branching morphogenesis in the pancreas while preserving the integrity of the acinar-ductal functional unit. Fine-tune regulation of the IGF/PI3K pathway regulates ductal cell rearrangements, enabling first clefting and splitting of the acini and then duct bifurcation.

## Introduction

Numerous epithelial organs undergo branching morphogenesis during their development to build complex, arborized networks and acquire specialized tissue architectures. Examples include the lung, kidney, and exocrine glands, such as the pancreas.^1^^,^^2^^,^^3^ Understanding the establishment and maintenance of tissue architecture is a central question in developmental biology with a direct implication on organ physiology and disease. In fact, disruption of these same mechanisms in an embryo can result in developmental diseases,^1^^,^^3^ while in adult life, loss of tissue architecture may occur at early stages and across diverse human cancers.^1^^,^^4^

Live imaging approaches combined with organ cultures and genetic perturbations have provided invaluable insights into the molecular and cellular basis of branching. Several signaling pathways and cellular mechanisms have been identified as conserved regulators of branching morphogenesis in diverse organs, while important organ-specific differences have also arisen.^1^^,^^4^ Recent studies have shown that each organ may employ a unique set of branching strategies to generate specialized shapes that are optimized for the organ’s physiological role.^1^^,^^3^^,^^5^^,^^6^ Thus, the challenge is to decipher both universally shared and unique mechanisms.

The pancreas is a highly branched organ, wherein groups of acinar cells cluster around terminal ducts to form functional exocrine secretory units, and ductal cells line the tubular network of the pancreatic duct system.^7^^,^^8^ In the mouse embryo, the formation of primary epithelial branches starts around embryonic day (E) 12.5, coinciding with the spatial segregation of pro-acinar and pro-ductal/endocrine-bipotent pancreatic progenitors.^8^^,^^9^^,^^10^ Subsequently, a luminal plexus is remodeled into a tubular network in the center of the pancreatic epithelium, which is the site of endocrine differentiation, while the periphery displays ramifying branches with acinar cells placed at the ends of terminal ducts.^11^^,^^12^^,^^13^^,^^14^^,^^15^ While early morphogenetic events underlying primary branching have been characterized to some extent,^16^^,^^17^^,^^18^^,^^19^^,^^20^ there is no understanding of how acinar and ductal cells subsequently rearrange, when branches bifurcate to form a ramifying tree and acini multiply. Several fundamental questions remain unanswered about the coupling of differentiation and morphogenesis. It is indeed unclear the degree to which the morphogenetic potential of the tissue is determined by its differentiation state because ductal-acinar units are already established while new branches are still being generated. Moreover, the molecular and cellular mechanisms ensuring branching morphogenesis while preventing detrimental ductal-acinar cell rearrangements are elusive.

Here, we addressed these questions by performing live imaging and quantitative analyses of the cellular events underlying branching in the mouse embryonic pancreas. We found that ductal cells drive branching morphogenesis by a “protrude and pull” mechanism, which is insulin growth factor (IGF)/phosphatidylinositol 3-kinase (PI3K) dependent. Our analyses of conditional *Igf1r* knockout mice and *ex vivo* pancreatic culture assays showed that ductal cell rearrangements are finely tuned by PI3K, enabling first clefting and splitting of the acini and then duct bifurcation. At the molecular level, PI3K activity depends on actomyosin contractility in ductal cells, highlighting an important role for tissue fluidity during branch formation in the pancreas. Together, our findings explain how acinar unit multiplication and branch bifurcation are synchronized in the developing pancreas. They also strongly suggest that differentiated acini need to be established before bifurcating branches can be formed.

## Results

### Ductal cells drive cleft-mediated branching morphogenesis in the pancreas

**Figure 1:**
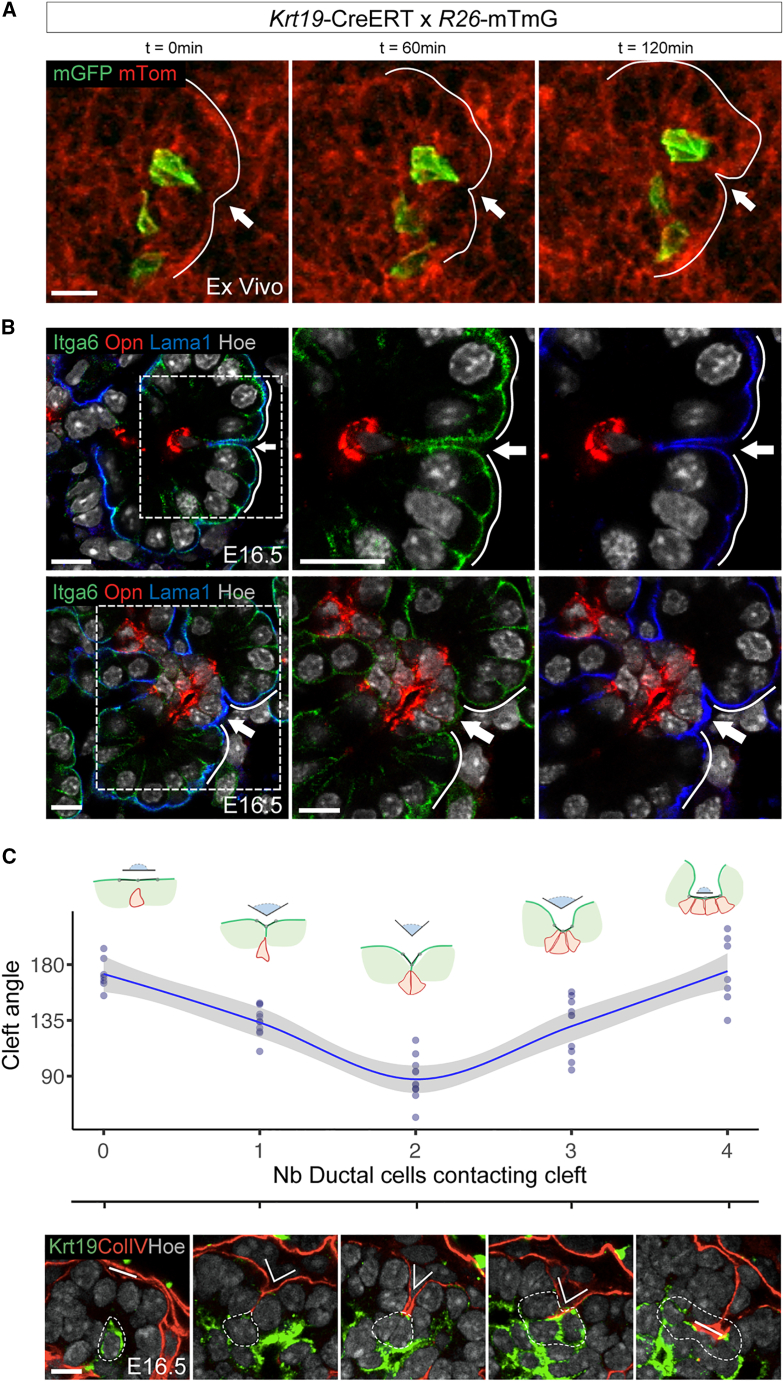
Pancreatic ductal cell protrusions promote cleft formation during branching morphogenesis (A) Representative confocal time-lapse images of *Krt19-*Cre^ERT^;*R26*mTmG pancreatic explants collected at E12.5 and cultured for 48 h in the presence of 4-hydroxytamoxifen (4-OHT). The number of ductal cells recombined represented on average 6.2% (SD = 0.79) of all epithelial cells. Membrane-bound GFP^+^ ductal cell (mGFP, green) is shown protruding between epithelial cells (red); arrow indicates a site of cleft formation. Time-lapse sequences of images from Video S1. Scale bars, 10 μm. (B) Representative confocal images of E16.5 pancreatic tissue immunostained for integrin alpha6 (Itga6), osteopontin (Opn), and laminin alpha-1 (Lama1). Top panel, arrow indicates a cleft with a ductal cell (Opn^+^) underneath in contact with the BM (Lama1^+^); bottom panel, arrow indicates an enlarged cleft with three Opn^+^ ductal cells underneath. Boxed regions are shown at a higher magnification with split channels on the right. n = 4. Scale bars, 10 μm. (C) Quantitative analysis of cleft progression. Top panel, plot showing the spanning angle of a cleft plotted against the number of underlying ductal cells (Krt19^+^) in direct contact with the BM (ColIV^+^). A local regression fitting a smooth line to the data is shown with a 0.95 confidence interval. Bottom panel, representative confocal images of E16.5 pancreatic tissue immunostained for cytokeratin19 (Krt19) and collagen IV (ColIV). Dotted lines delineate Krt19^+^ ductal cells, while solid lines indicate the cleft spanning angle demarcated by the acinar BM (ColIV^+^). n = 3 whole-mount pancreata. Scale bars, 10 μm.

Here, we sought to elucidate the cellular mechanisms by which cells rearrange to support pancreas branching morphogenesis. To gain insight into these dynamic events, we first performed time-lapse imaging of ductal cells in mouse pancreatic explants collected at E12.5 (Figure 1A). *Ex vivo* pancreatic cultures closely recapitulate the *in vivo* morphogenesis processes, providing a simple platform for their observation (Figures S1A and S1B).^21^^,^^22^ We used the tamoxifen-inducible transgenic (Tg) cytokeratin 19 (*Krt19*)-CreERT line^23^ in combination with the Tg(mTmG) fluorescent reporter line^24^ to mosaically label and track ductal cells in pancreatic explants. We observed that the Krt19-mG ductal cells that localized in the centroacinar space often extend cellular protrusions between mT^+^ epithelial cells and direct them toward the basement membrane (BM) (Figure 1A; Video S1). Notably, our time-lapse imaging showed that these ductal protrusions are dynamic; upon multiple cycles of contact formation and retraction, they eventually pull the BM between neighboring cells, initiating the formation of epithelial clefts (Figures 1A and S1C; Video S1). Of all the tracked mG^+^ ductal cells, 13.8% (n = 144 cells tracked; SD = 6.0) formed protrusions toward the BM, of which 30.6% (SD = 2.4) were in contact with a forming cleft (Video S1).

Video S1. Ductal cells extend protrusions in between pancreatic epithelial cells, related to Figure 1 Confocal time-lapse video of a *Krt19-Cre*^*ERT*^*; R26mTmG* pancreatic explant collected at E12.5 and imaged after 48h of 4-Hydroxytamoxifen (4OHT) treatment. mGFP+ ductal cells (green) are shown extending protrusions in between pancreatic epithelial cells (red). The first protrusion (first arrow) does not lead to epithelial clefting, while the second one does (second arrow). White line delineates the basal side of acinar cells, where the basement membrane (BM) lies.

To further assess whether the retraction of ductal protrusions might induce cleft formation, we performed immunofluorescence (IF) staining for markers of acinar (integrin alpha6, amylase, and Cpa1); duct (osteopontin, Sox9, mucin, and Krt19); and BM (laminin1) on pancreas tissue sections at E16.5, when many clefts are visible (Figures 1B and S1D). In all the examined acinar clusters, we systematically found that below each cleft was positioned at least one ductal cell with a protrusion anchored to the acinar BM, which was invaginated between two acinar cells (Figure 1B). Specifically, in E16.5 pancreatic tissue sections, 6.15% of ductal cells (n = 954 cells analyzed; SD = 2.7) were seen in contact with a cleft, and clefts were observed in 33.3% of the acini (n = 94; SD = 5.6) (Figure 1B).

In addition, by measuring the cleft spanning angle, we observed that the angle is narrower when at least one ductal cell is in contact with the BM, whereas it becomes wider when more than two ductal cells are recruited underneath (Figure 1C; Video S2). This suggests that multiple ductal cells need to contact a cleft in order to enlarge it, and they might act synergistically to stabilize it. During the cleft progression, we observed that terminal ductal cells change shape, from a tear-drop shape into a cuboidal shape, and rearrange into a single-layer epithelium, which separates the newly formed acini (Figures 1B and S1C). Finally, our temporal analysis of pancreatic epithelium showed that cleft-ductal structures are visible from E14.5 onward (Figure S1E), which coincides with the period of secondary branching expansion.^20^^,^^22^

Video S2. Ductal cells underneath clefts, related to Figure 1 Light-sheet fluorescent microscopy images of a ductal branch underlying acini from E16.5 pancreas immunostained for ColIV (red) and Krt19 (green). Part 1: 3D rendering of the tissue, Krt19 isosurfaced in green. First arrow indicates the initiation of a cleft; second arrow points at an enlarged cleft, which separates two acini. Part 2: Z-stack visualization of the area with the enlarged cleft (third arrow).

The BM is known to exert both biochemical and biophysical roles during branching morphogenesis in different organs.^6^ To assess any BM-dependent role in clefting, we treated the pancreatic explants with type IV collagenase to digest BM components. This treatment strongly reduced cleft numbers without affecting acinar cell survival (Figures S1F–S1H), highlighting the importance of BM integrity for ductal cells to drive clefting. Moreover, by analyzing the BM deposition around the acini, we noted an inverse correlation between acinar BM thickness and the acinus size, with the bigger acini being surrounded by a thinner BM (Figures S1I–S1J).

**Figure 2:**
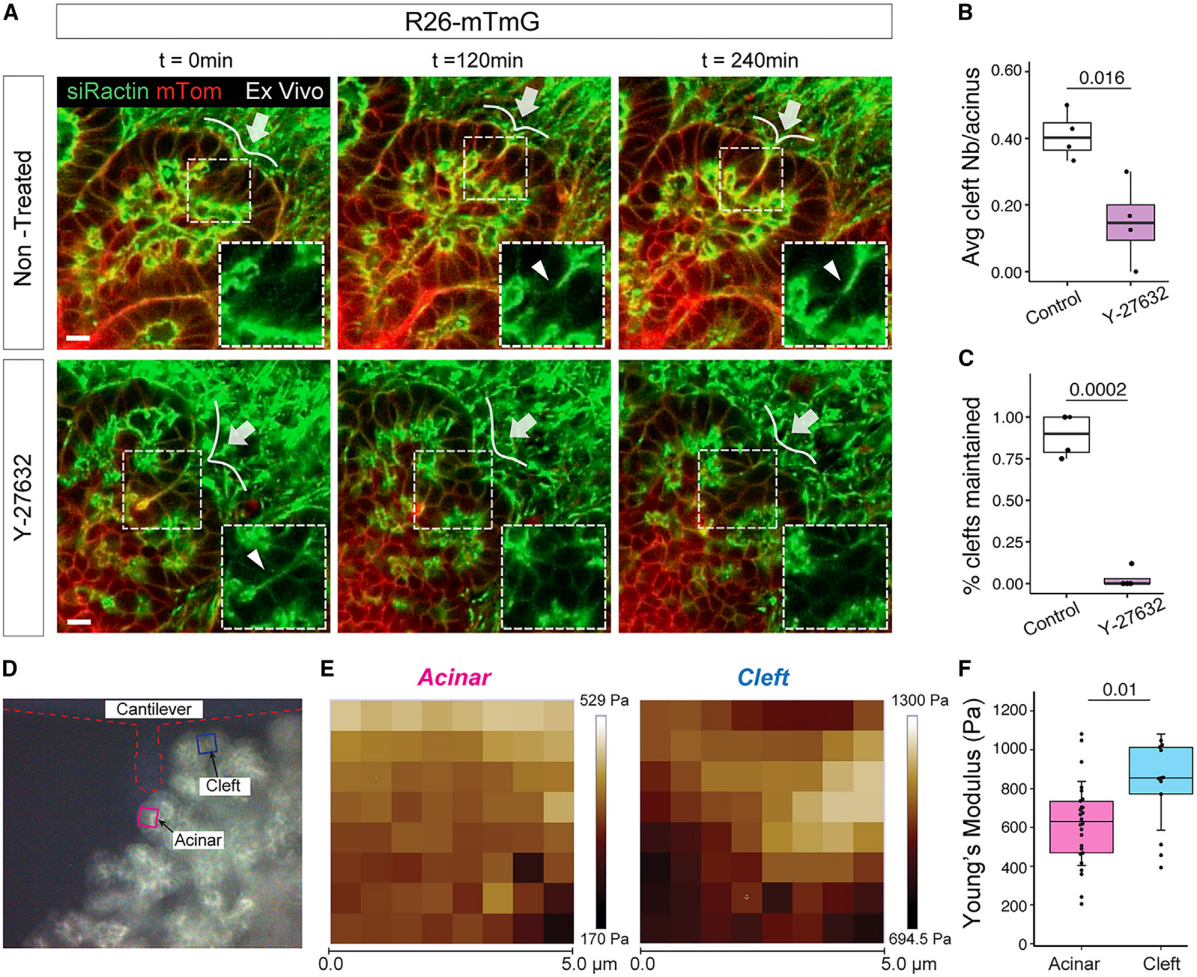
Cleft formation and maintenance depend on actomyosin contractility (A) Representative confocal time-lapse images of *R26-*mTmG pancreatic explants collected at E12.5 and cultured for 48 h. Non-treated control (top panel) explants or treated with Y-27632 (bottom panel) were imaged for 15 h. SiR-actin (green) marks F-actin, while membrane-bound Tomato protein (mT) (red) marks pancreatic epithelial cells. Arrows indicate the site of formation of a cleft. Insets show higher magnification of the boxed regions, displaying the separated siR-actin channel; arrowheads indicate the siR-actin accumulation in the forming cleft. Time-lapse sequences of images taken from Video S3 (non-treated) and Video S5 (Y-27632). Scale bars, 10 μm. (B) Quantification of cleft number in non-treated pancreatic explants or upon Y-27632 treatment. The average number (Nb) of clefts is shown relative to the total number of acini analyzed. n = 4 explants per treatment. Student’s t test. (C) Quantification of clefts present at the start of the time-lapse acquisition and maintained during the acquisition period (15 h) in non-treated and Y-27632-treated explants. n = 4 explants per treatment. Student’s t test. (D) Representative image of a *Pdx1-*Cre;*R26*mTmG pancreatic explants collected at E12.5 and cultured for 72 h. Boxed regions show two 5 μm × 5 μm representative acinar (magenta) and cleft (blue) sites whose stiffness was measured with the AFM. The AFM cantilever contour is outlined with a red dashed line. (E) Representative Young’s modulus maps for an acinar (left) and cleft (right) region. Each square corresponds to the Young’s modulus obtained from one force-distance curve. (F) Young’s modulus (Pa) of the acinar (magenta) and cleft (blue) regions. Each point corresponds to the average Young’s modulus of one 5 μm × 5 μm region, typically having 40–50 measurements. n = 26 (acinar); n = 12 (cleft) regions from 7 different pancreatic explants. Two-tailed unpaired Student’s t test.

To investigate whether cytoskeletal contraction is involved in ductal-mediated clefting, we performed time-lapse imaging of Tg(mTmG) pancreatic explants using the siR-actin dye to visualize actin dynamics during clefting. We observed that actin is consistently recruited to the basal side of ductal cells as clefts form (Figure 2A; Video S3). Next, to determine if actomyosin contractility is required for cleft formation, we treated pancreatic explants with the Rho Kinase (ROCK) activity inhibitor Y-27632 and quantified the number of clefts over the duration of the time-lapse acquisition. ROCK inhibition not only significantly decreased the frequency of cleft formation (Figure 2B) but also impaired the maintenance of clefts, which were previously formed and present at the beginning of the time-lapse acquisition (Figure 2C; Videos S4 and S5). Together, these results suggest that proper cytoskeleton contraction is required for cleft initiation and must be maintained in ductal cells for a certain duration to ensure cleft stabilization and prevent the split acini from merging.

Video S3. Accumulation of siR-actin in the forming cleft, related to Figure 2 Confocal time-lapse video of *R26mTmG* pancreatic explants cultured with siR-actin and imaged for 15h. In red are mTom^+^ epithelial cells and in green is SiR-actin, a F-actin labelling probe. Arrows indicate the initiation of a cleft and the localization of siR-actin in the forming cleft.

Video S4. Decreased frequency of cleft formation in the presence of the ROCK inhibitor Y-27632, related to Figure 2 Confocal time-lapse video of *R26mTmG* pancreatic explants cultured with siR-actin and imaged for 15h after Y-27632 exposure. In red are mTom^+^ epithelial cells and in green is SiR-actin, a F-actin labelling probe. No clefting is observed upon ROCK inhibition by Y-27632.

Video S5. Impaired cleft maintenance in the presence of the ROCK inhibitor Y-27632, related to Figure 2 Confocal time-lapse video of *R26mTmG* pancreatic explants cultured with siR-actin and imaged for 15h after Y-27632 exposure. In red are mTom^+^ epithelial cells and in green is SiR-actin, a F-actin labelling probe. Arrows indicate the loss of a cleft upon ROCK inhibition by Y-27632.

Next, we used atomic force microscopy (AFM) to assess the mechanical properties of the acinar/tip and cleft sites in embryonic pancreatic explants and determine whether differences in stiffness could be observed. To specifically probe the properties of epithelial cells at different locations, we applied AFM in combination with fluorescence imaging to live explants from *Pdx1-Cre*;mTmG Tg embryos (Figure 2D). Interestingly, our data showed that the Young’s modulus in the cleft sites is significantly higher than that in the acinar/tip sites (∼900 vs. ∼600 Pa) of the explants (Figures 2D–2F), suggesting an increase in tension at cleft sites. This is in line with the actin accumulation and actomyosin-based contractility mechanism operating in ductal cells (Figures 2A–2C). Taken together, these findings are consistent with a model in which pancreatic terminal ductal cells, through an actomyosin-dependent “protrude and pull” mechanism, drive ductal-mediated clefting and split acini in two.

### PI3K regulates cleft formation and branching morphogenesis

**Figure 3:**
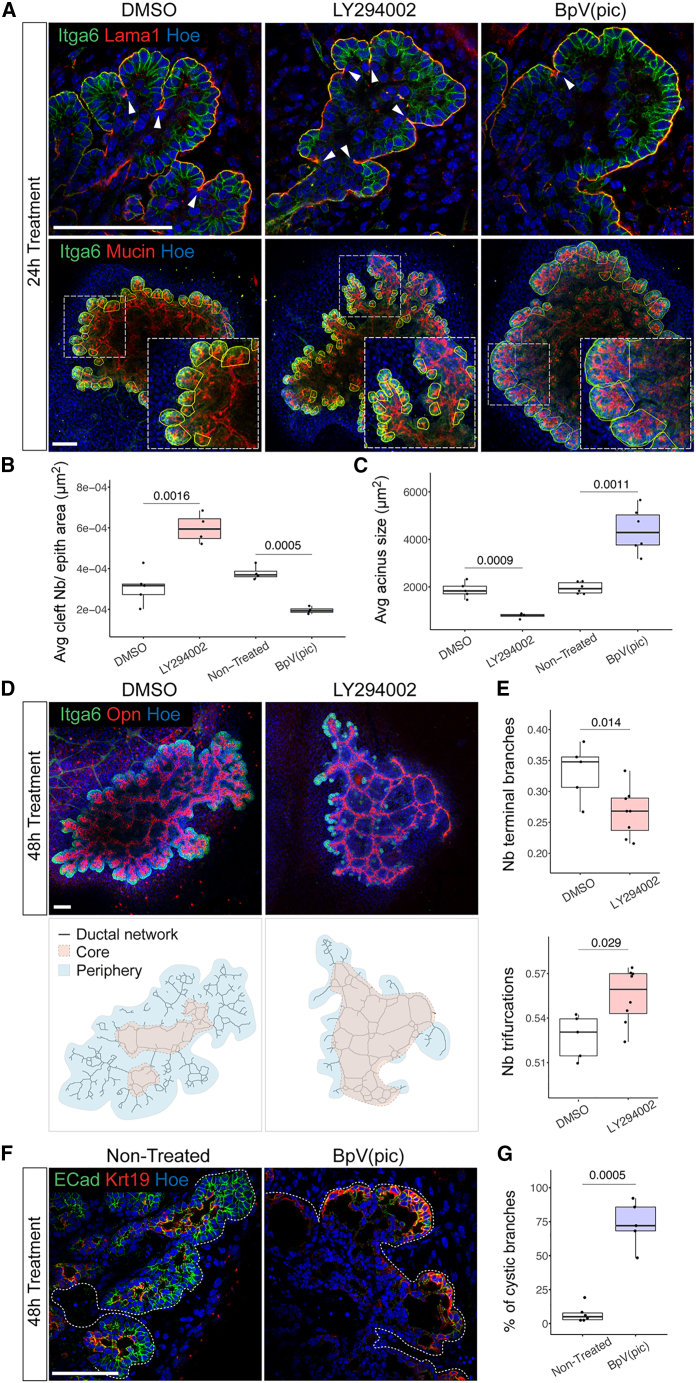
PI3K pathway regulates cellular rearrangements underlying branching morphogenesis (A) Representative confocal images of pancreatic explants collected at E12.5, cultured for 24 h in the presence of DMSO (control), LY294002, or BpV(pic) and immunostained with indicated antibodies. Top: arrowheads indicate clefts. Bottom: acini are delineated with a yellow line, and insets show higher magnifications of boxed regions. Scale bars, 100 μm. (B) Quantification of clefts (number per μm^2^) in pancreatic explants upon indicated treatments. The number (Nb) of clefts are shown relative to the area of the pancreatic epithelium (in μm^2^). n = 4–5 explants per treatment. Student’s t tests. (C) Quantification of acini size (in μm^2^) in pancreatic explants upon indicated treatments. n = 4–6 explants per treatment. Student’s t tests. (D) Top panel: representative confocal images of pancreatic explants cultured for 48 h with DMSO (Control) or LY294002 and immunostained for Itga6 and Opn. Scale bars, 100 μm. Bottom panel: binary ductal networks extracted from confocal images of the explants shown in top panel. Blue and beige overlays delineate the ductal network core and periphery regions, which are composed of interconnected and terminal ducts, respectively. (E) Quantification of terminal ends (top) and trifurcations (bottom) in the ductal network of untreated and treated explants, expressed relative to the total number of branches. n = 5–8 explants per treatment. Student’s t tests. (F) Representative confocal images of pancreatic explants either non-treated controls or explants treated with BpV(pic) for 24 h and immunostained for E-cadherin (ECad) and Krt19. White dotted lines mark the pancreatic epithelium. Scale bars, 100 μm. (G) Quantification of terminal cystic dilations relative to the total number of branches. n = 5–6 explants per treatment. Student’s t test.

The PI3K pathway is well known for regulating the ability of epithelial cells to protrude and rearrange during morphogenesis.^25^^,^^26^^,^^27^^,^^28^ To investigate the potential influence of PI3K on cell rearrangement and ductal-mediated clefting during pancreatic morphogenesis, we treated explants collected at E12.5 either with the PI3K antagonist, LY294002, or the agonist, BpV(pic). After 24 h of treatment, PI3K inhibition significantly increased the number of epithelial clefts, whereas PI3K overactivation decreased their number (Figures 3A and 3B). Notably, LY294002-induced clefting was accompanied by acini fragmentation, as evidenced by their significant decrease in size and increase in number (Figures 3A, 3C, and S2A), while the total acinar cell number remained unaffected (Figure S2A). Conversely, PI3K overactivation caused an enlargement of the acini (Figures 3A and 3C). The morphological changes caused by PI3K dysregulation were not related to a change in cell proliferation because the number of phospho-Histone 3 (PH3)^+^ cells was not significantly different in explants treated with LY294002 or BpV(pic) compared with non-treated controls (Figures S2B and S2C). High acinar cell death rate was detected in explants upon LY294002 treatment, which did not significantly impact acinar cell number after 24 h of treatment but impaired explant growth after 48 h (Figures S2A, S2C, S2D, and S2G).

During pancreas development, the ductal network grows following two distinct but concomitant morphogenetic programs. In the center (a.k.a*.* core), an expanding ductal mesh remodels to form a network of interconnected ducts, whereas in the periphery, terminal ducts grow and branch into a ramified network.^12^^,^^29^ Upon long-term exposure to LY294002, we observed a major impact on the ductal network at the periphery, displaying a striking reduction of ramified branches compared with the core area. To quantify this change in architecture, we extracted the ductal network topologies of the explants^30^ (Figure 3D). Our quantitative analysis showed that PI3K inhibition strongly limits branching morphogenesis at the periphery, with many ductal branches being devoid of acini, while central ducts remodeling in the core expanded (Figures 3D and 3E). By tracking the transformation of the clefts over 48 h time course experiments, we found that LY294002 treatment prevented the stabilization of clefts into branch bifurcations (Figures S2E and S2F). Together, our results indicate that prolonged PI3K inhibition induces first uncontrolled clefting and over-fragmentation of the acini, which subsequently leads to acinar loss and reduction of secondary and terminal branches. Conversely, prolonged overactivation of PI3K transformed enlarged acini into cysts solely composed of ductal cells, as judged by the expression of Krt19 (Figures 3F and 3G).

### Igf1r acts upstream of PI3K in ductal cells to regulate branching

**Figure 4:**
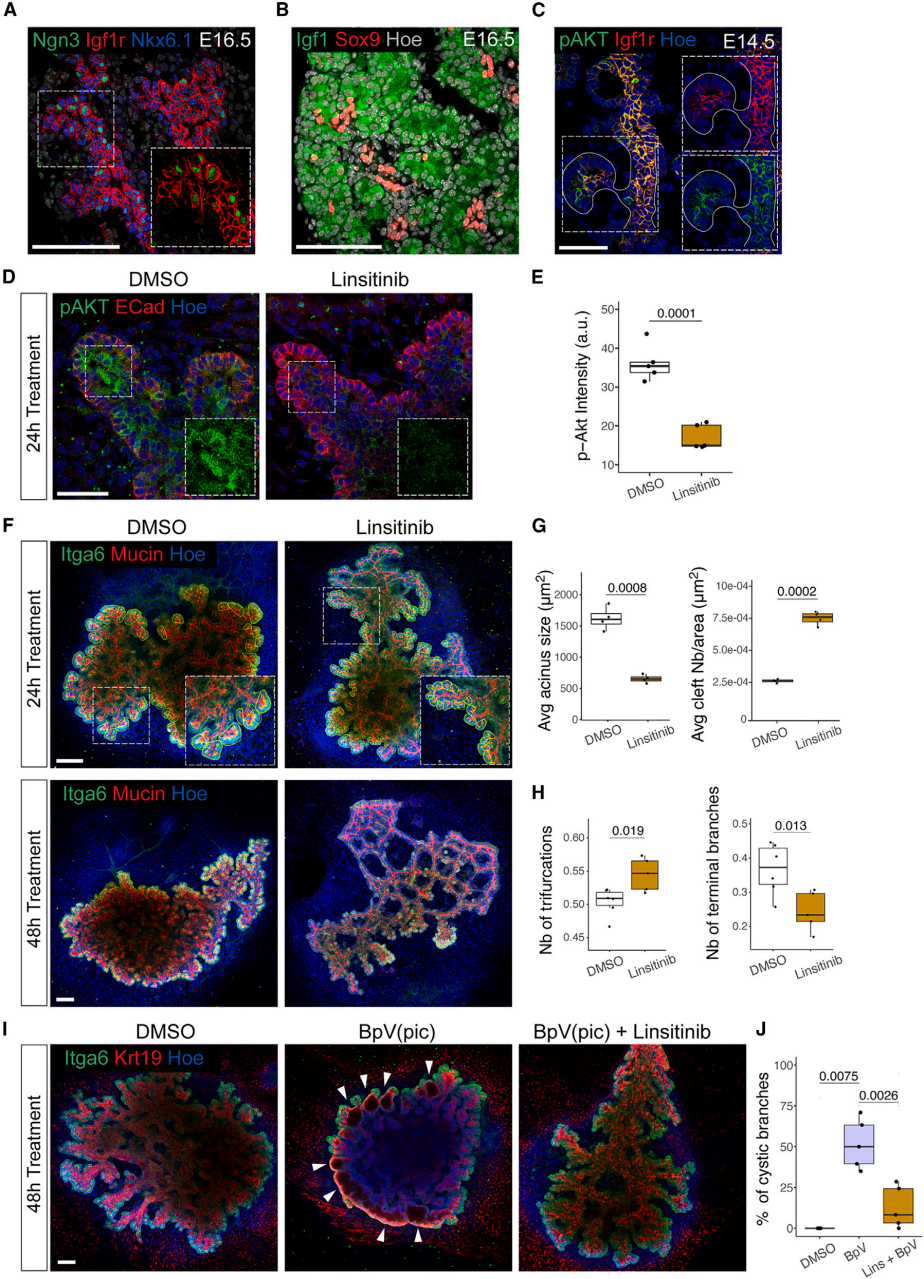
IGF signaling acts upstream of the PI3K pathway to regulate branching morphogenesis and acinar fragmentation (A) Representative confocal image of pancreatic tissue at E16.5 immunostained for Neurogenin3 (Ngn3), Igf1r, and Nkx6.1. Inset shows higher magnifications of boxed region with Nkx6.1 excluded. Scale bars, 100 μm. (B) Representative confocal image of pancreatic tissue at E16.5 immunostained for Igf1 and Sox9. Scale bars, 100 μm. (C) Representative confocal image of pancreatic tissue at E14.5 immunostained for phosphorylated Akt (pAKT) and Igf1r. Insets show higher magnifications of the boxed regions as separated channels. Scale bars, 50 μm. (D) Representative confocal images of pancreatic explants collected at E12.5, treated for 24 h with DMSO (control) or linsitinib and immunostained for pAKT and ECad. Insets show higher magnifications of the boxed regions, pAKT channel only. Scale bars, 50 μm. (E) Quantification of pAKT intensity in ECad+ pancreatic explants. n = 5 explants per treatment. Student’s t tests. (F) Representative confocal images of pancreatic explants collected at E12.5, treated for 24 h (top panel) or 48 h (lower panel) with DMSO (Control) or linsitinib, and immunostained for Itga6 and mucin. Top panels: insets show higher magnifications of boxed regions; yellow lines delineate acini. Scale bars, 100 μm. (G) Quantification of acini size (left panel) and clefts number (Nb) (right panel) (in μm^2^) in pancreatic explants treated for 24 h with DMSO (Control) or linsitinib. Clefts Nb are shown relative to the area of the pancreatic epithelium (in μm^2^). n = 4 explants per treatment. Student’s t tests. (H) Quantification of trifurcations (left) and terminal ends (right) in the ductal network of explants treated for 48 h with DMSO (control) or linsitinib, expressed relative to the total number of branches. n = 5–7 explants per treatment. Student’s t tests. (I) Representative confocal images of pancreatic explants collected at E12.5; treated for 48 h with DMSO (control), BpV(pic), alone, or in combination with linsitinib; and immunostained for Itga6 and Krt19. Arrowheads indicate enlarged acini resembling cystic structures. Scale bars, 100 μm. (J) Quantification of terminal cystic dilations relative to the total number of branches. n = 5 explants per treatment. Mann-Whitney U test (DMSO vs. BpV) and Student’s t test (BpV vs. Lins + BpV).

The PI3K pathway can be activated by a variety of ligands upon binding to receptor tyrosine kinases (RTKs) receptors at the cell membrane.^28^ In order to identify which signals trigger PI3K activation in ductal cells, we analyzed the expression of RTK receptors in a publicly available single cell RNA sequencing (scRNA-seq) dataset of pancreatic epithelial cells between E12.5 and E18.5.^31^ We identified *Igf1r* as a promising candidate because it showed high levels of expression in the ductal cell cluster (Figures S3A and S3B). IF analysis confirmed the remarkably specific localization of Igf1r in ductal cells at E12.5, E14.5, and E16.5 (Figures 4A and S3D). Conversely, *Igf1* ligand showed abundant and increasing levels of expression in acinar cells from E12.5 to E18.5 (Figures 4B and S3A–S3C). This suggests that acinar cells can send a short-range signal to adjacent ductal cells and that the strength of this signal might increase during pancreas development. Notably, the accumulation of strong and specific phospho-AKT staining in ductal cells, as shown by IF at E14.5 (Figure 4C), indicated downstream activation of the PI3K pathway.

3D imaging by light-sheet fluorescence microscopy enabled us to clearly visualize Igf1r membrane localization in ductal cells, decorating the protrusions; their intimate connection with the initiating clefts (Figure S3F; Video S6); and transient recruitment of phospho-myosin (pMyo) (Figure S3E).

Video S6. Igf1r membrane localization in ductal cells, related to Figure 4 3D rendering of E16.5 pancreatic tissue immunostained for Igf1r (red) and Laminin-alpha1 (Lama1) (green) and imaged by light-sheet fluorescent microscopy. Selected region of interest displaying the organization of the Igf1r^+^ ducts (red isosurface) with respect to the acini (Lama1^+^ BM, green isosurface). Nuclei were stained with Hoechst (blue).

**Figure 5:**
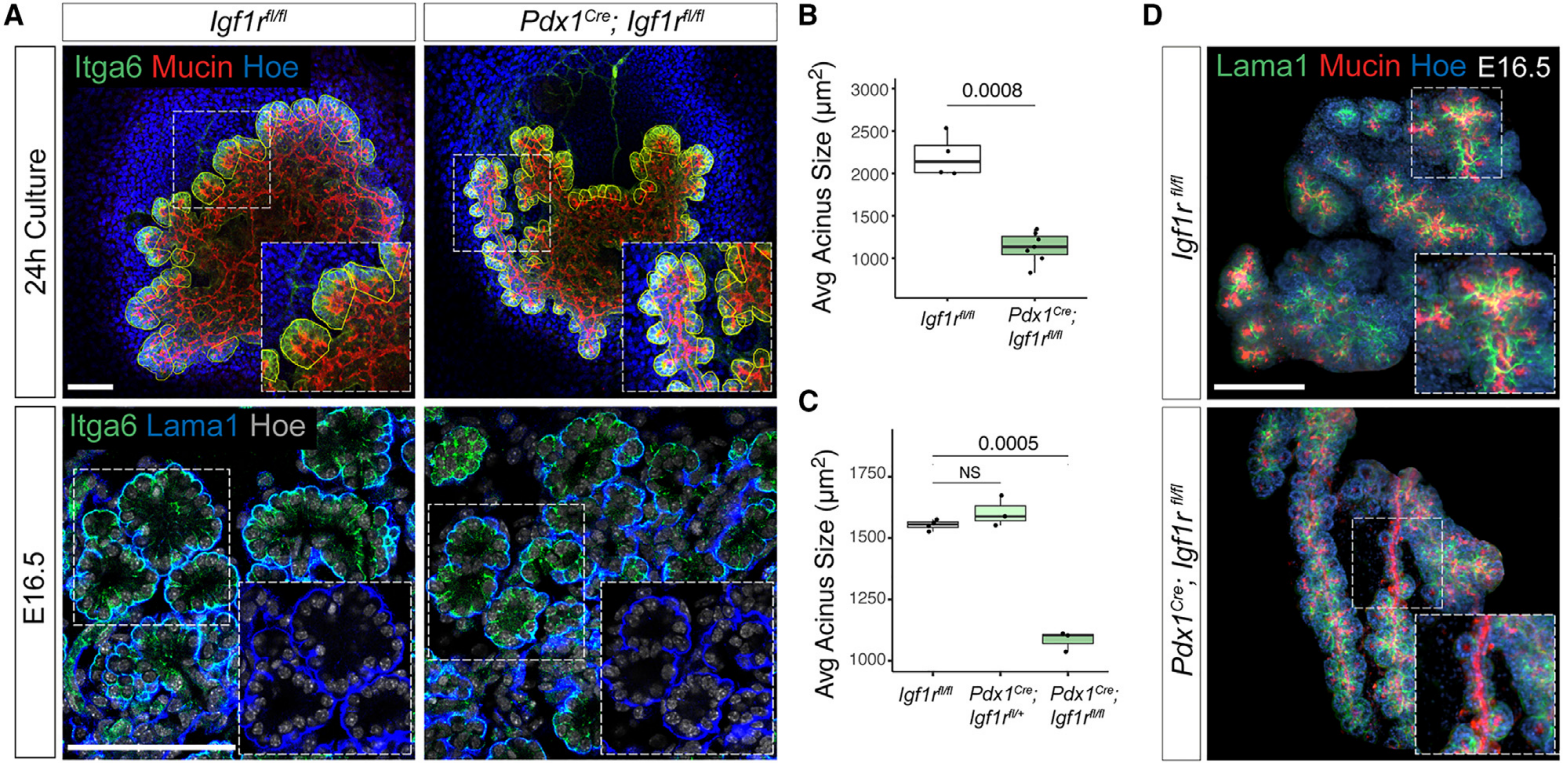
IGF/PI3K regulates branching morphogenesis and acinar fragmentation *in vivo* (A) Representative confocal images of *Igf1r*^*flox/flox*^ and *Pdx1-*Cre;*Igf1r*^*flox/flox*^ pancreatic explants (top panel) and E16.5 pancreatic tissue (bottom panel). Explants were immunostained for Itga6 and mucin; pancreatic tissue for Itga6 and Lama1. Yellow lines delineate acini; insets show higher magnifications of boxed regions. Scale bars, 100 μm. (B) Quantification of acini size (in μm^2^) in *Igf1r*^*flox/flox*^ and *Pdx1-*Cre;*Igf1r*^*flox/flox*^ pancreatic explants. n = 4–7 explants per genotype. Student’s t test. (C) Quantification of acini size (in μm^2^) in *Igf1r*^*flox/flox*^, *Pdx1-*Cre;*Igf1r*^*flox/+*^ and *Pdx1-*Cre;*Igf1r*^*flox/flox*^ E16.5 pancreatic tissue. n = 3–5 embryos per genotype. Student’s t test. (D) Representative light-sheet microscopy images of E16.5 *Igf1r*^*flox/flox*^ and *Pdx1-*Cre;*Igf1r*^*flox/flox*^ pancreata immunostained for Lama1 and mucin. Insets show higher magnifications of boxed regions highlighting unbranched ductal segments and partial loss of acini in the mutant pancreas. Scale bars, 1 mm.

Next, to functionally probe whether Igf/Igf1r signaling is responsible for PI3K activation in ductal cells, we inhibited the pathway activity in pancreatic explants using linsitinib, an Igf1r antagonist (Figures 4D and 4E). The blockade of Igf1r signaling led to a significant increase in acinar fragmentation and cleft number (Figures 4F and 4G). However, this was followed by a strong defect in the number of clefts that were stabilized into branch bifurcations, resulting in an overall reduction of secondary branches at the periphery after 48 h (Figures 4F, 4H, S3H, and S3I). Similar to the LY294002 treatment, linsitinib increased acinar cell death and impaired explants growth after 48 h (Figures S3G and S3J). Overall, the linsitinib treatment remarkably phenocopied PI3K inhibition, suggesting that Igf/Igf1r mediates the interaction between acinar and terminal ductal cells. To further test this hypothesis, we performed rescue experiments by blocking Igf1r activities in pancreatic explants exposed to the PI3K agonist, BpV(pic). Notably, the simultaneous treatment with linsitinib and BpV(pic) led to a significant decrease in the formation of cysts in comparison with explants treated with BpV(pic) alone (Figures 4I and 4J), indicating that Igf/Igf1r signals act upstream of PI3K activation in ductal cells. In addition, we undertook a genetic approach to specifically knockout *Igf1r*^32^ in the mouse pancreas epithelium (Figures S4A and S4B). *Igf1r* deletion in *Pdx1*-Cre;*Igf1r*^*flox/flox*^ pancreatic explants caused a fragmentation of the acini *ex vivo* (Figures 5A and 5B), similar to that observed after LY294002 and linsitinib treatments (Figures 3A and 4F), as well as *in vivo* in pancreatic tissue at E16.5 (Figures 5A and 5C). This acinar phenotype was coupled with defects in the bifurcation and ramification of the secondary ductal branches in the absence of severe pancreas growth defects (Figures 5D and S4C). On closer inspection, the mutant pancreata displayed long and unramified ductal segments that were sometimes devoid of acini (Figure 5D). Overall, our results underscore a role for IGF signaling in regulating pancreas branching morphogenesis. Notably, the double-knockout embryos for *Igf1r* and the highly homologous family member, *Insr*, have been previously reported to display stronger defects in pancreatic growth and branching morphogenesis,^33^ suggesting functional compensation of the *Insr* in *Igf1r* mutant pancreas. Consistently, we also observed that the linsitinib treatment, which targets both *Igf1r* and *Insr*, leads to a more severe phenotype, including increased acinar cell death, compared with the *Igf1r* deletion alone (Figures S3G and S4A).

### PI3K finely tunes ductal tissue fluidity to enable branching morphogenesis

**Figure 6:**
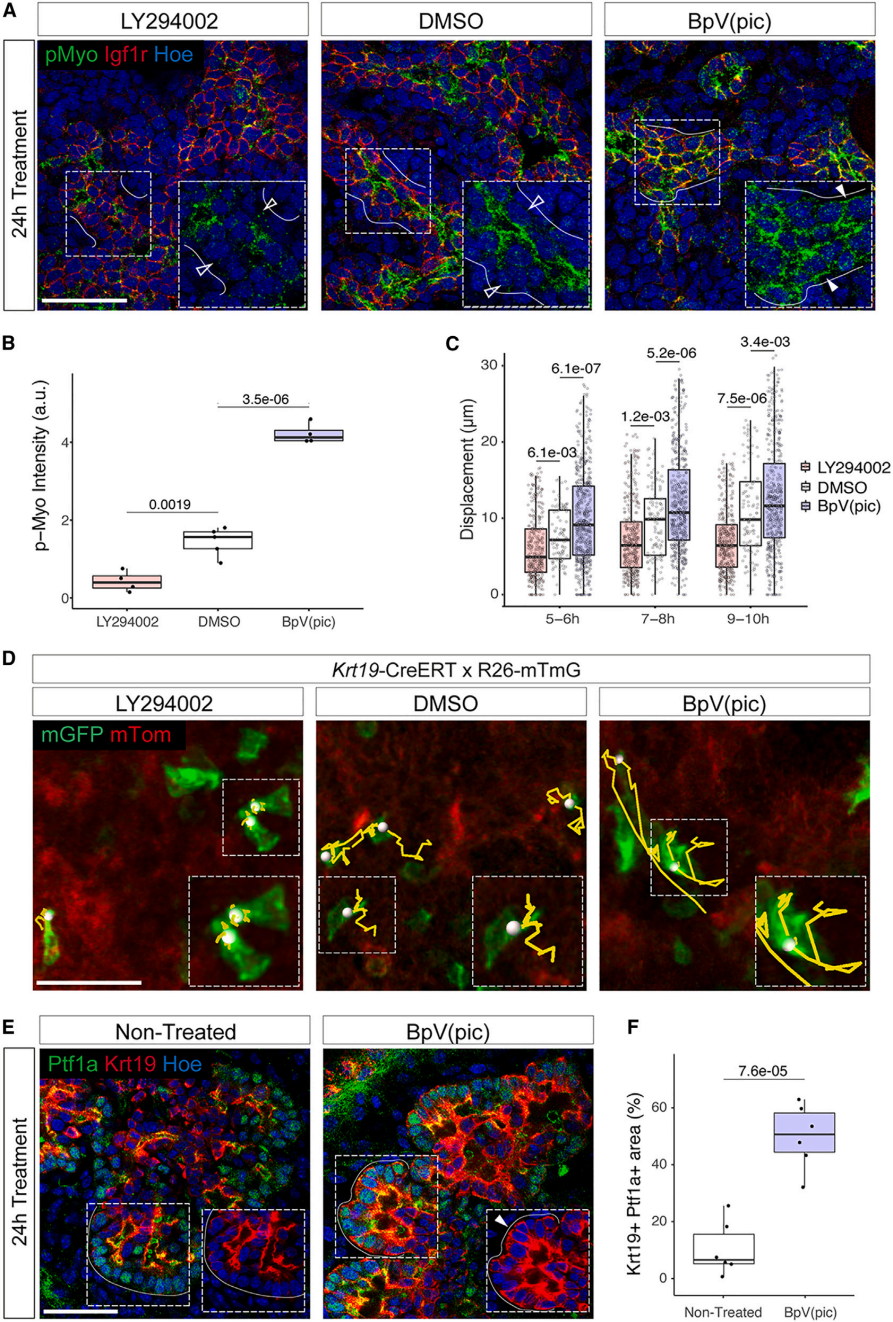
The PI3K pathway regulates ductal tissue fluidity in an actomyosin-dependent manner (A) Representative confocal images of cryosections of pancreatic explants collected at E12.5; treated 24 h with DMSO (control), LY294002, or BpV(pic); and immunostained for pMyo and Igf1r. Insets show higher magnifications of the boxed regions, displaying the separated pMyo channel. White lines delineate the basal side of ducts; filled arrowheads (BpV(pic) condition) indicate pMyo relocalization on the basal side of Igf1r^+^ ductal cells, while empty arrowheads show absence of pMyo on the basal side of ductal cells in the DMSO (control) and LY294002 conditions. Scale bars, 50 μm. (B) Quantification of pMyo intensity at ductal cell membranes in pancreatic explant cryosections. n = 4–5 explants per treatment. Student’s t tests. (C) Quantification of ductal cell displacement (μm) in explants upon indicated treatments during a 2-h interval, ranging from 5 to 10 h post-acquisition start. The displacement of hundreds of cells from n = 3 explants (per treatment) was analyzed. Student’s t tests. (D) Representative confocal time-lapse images of *Krt19-*Cre^ERT^*;R26mTmG* pancreatic explants collected at E12.5; cultured for 48 h with 4-OHT; treated with DMSO (control), LY294002, or BpV(pic); and recorded for 12 h. Representative tracks of mGFP+ ductal cells (green) taken from t = 4 h to t = 8 h frames of the time-lapse are shown in yellow. Scale bars, 50 μm. (E) Representative confocal images of pancreatic explants non-treated or treated with BpV(pic) and immunostained for Ptf1a and Krt19. Insets show the boxed regions with Ptf1a excluded. White lines delineate the basal surface of acinar cells; arrowhead indicates Krt19 localization in Ptf1a^+^ cells. Scale bars, 50 μm. (F) Quantification of the area occupied by Ptf1a^+^Krt19^+^ cells in pancreatic explants upon indicated treatments relative to the total epithelial area (shown in %). n = 6 explants per treatment. Student’s t test.

To mechanistically understand how the IGF/PI3K pathway regulates ductal cell rearrangement to drive pancreas branching morphogenesis, we analyzed the organization and dynamics of the actomyosin cytoskeleton upon LY294002 and BpV(pic) treatments. We observed that PI3K inhibition significantly reduced pMyo intensity in ductal cells, whereas PI3K overactivation increased it and relocalized it all around the cells (Figures 6A and 6B). IF for F-actin showed similar results (Figures S5A and S5B). This was specific to ductal cells and was not observed in acinar cells (Figure S5C).

Changes in actomyosin intensity and localization may translate into changes in tissue fluidity, which refers to the ability of cells to move and exchange neighbors within an epithelium.^34^ By contrast, in solid-like tissues, neighbors are maintained, and rearrangements are minimal.^34^ To assess whether PI3K might influence ductal tissue fluidity, we followed ductal cell displacement using live imaging analysis in pancreatic explants upon exposure to LY294002 or BpV(pic). Cell-tracking analysis showed that PI3K inhibition significantly impaired ductal cell displacement; many static cells that displayed columnar shape and strong apical constriction were detected (Figures 6C and 6D; Video S7). However, the LY294002 treatment did not affect protrusions formation (Figure S5D); therefore, ductal cells were still able to trigger initial cleft formation but not to subsequently rearrange and enlarge the clefts. On the other hand, PI3K overactivation increased ductal cell displacement (Figures 6C and 6D; Video S7); many ductal cells showed long-range migration, as in a fluid-like state. These results suggest that such uncontrolled ductal motility is responsible for the decreased ability of ductal cells to form clefts and for the disruption of acinar architecture (Figures 3A and 3F). In sum, ductal cell rearrangements appear to be finely tuned by PI3K, which enables cleft enlargement and duct bifurcation.

Video S7. PI3K modulates pancreatic ductal cell displacement, related to Figure 6 Confocal time-lapse video of *Krt19-Cre*^*ERT*^*;R26mTmG* pancreatic explants cultured for 48h with 4-OHT and imaged for 12h after BpV(pic) exposure. Tracks of mGFP+ ductal cells (green) from t=4h to t=8h time-lapse interval are displayed. Tracks are color-coded to illustrate cell displacement (in μm) from starting position. In red are mTom^+^ unrecombined epithelial cells.
Confocal time-lapse video of *Krt19-Cre*^*ERT*^*;R26mTmG* pancreatic explants cultured for 48h with 4-OHT and imaged for 12h after LY294002 exposure. Tracks of mGFP^+^ ductal cells (green) from t=4h to t=8h time-lapse interval are displayed. Tracks are color-coded to illustrate cell displacement (in μm) from starting position. In red are mTom^+^ unrecombined epithelial cells.
Confocal time-lapse video of *Krt19-Cre*^*ERT*^*;R26mTmG* pancreatic explants cultured for 48h with 4-OHT and imaged for 12h after BpV(pic) exposure. Tracks of mGFP+ ductal cells (green) from t=4h to t=8h time-lapse interval are displayed. Tracks are color-coded to illustrate cell displacement (in μm) from starting position. In red are mTom^+^ unrecombined epithelial cells.

In addition, we found that these morphological changes were accompanied by altered acinar cell fate. After 24 h of BpV(pic) treatment, a subset of Ptf1a^+^ acinar cells started to co-express ductal markers, such as Krt19, in the absence of acinar apoptosis (Figures 6E, 6F, and S2C). Subsequently, after 48 h, acinar cell markers were lost or strongly reduced at the tips of the explants (Figures S5E and S5F), while ectopic expression of Krt19 was maintained (Figures 6E and 6F), suggesting that PI3K overactivation triggers an acinar-to-ductal transdifferentiation to some extent.^35^^,^^36^ By contrast, we observed an increase in endocrinogenesis in LY294002-treated explants (Figures S5G and S5H). This increase might be linked to the expansion of the core ductal network (Figure 3D) as it has been shown in other studies.^12^^,^^15^^,^^16^

Overall, our results highlight that the PI3K-mediated regulation of ductal fluidity has a simultaneous impact on branching morphogenesis and pancreatic cell fate acquisition.

## Discussion

Clefting at the branch tips in most branched organs is regulated by extrinsic mechanisms, including local physical constraints by external cell compression or extracellular matrix (ECM) remodeling.^6^^,^^37^ Our study offers a different perspective on pancreatic morphogenesis, whereby tissue fluidity would allow ductal cells to actively remodel the acinar compartment, pointing to an epithelial-intrinsic mechanism. We propose a two-step model of pancreatic branching morphogenesis, subsequent to the patterning of acinar and ductal domains in primary branches: (1) formation of clefts by ductal cell protrusions and (2) ductal cells rearrangement into secondary branches. Overall, such a mechanism would enable the spatiotemporal synchronization between acini multiplication and ductal bifurcation, ensuring that acini remain connected to the terminal end of ramifying ducts. Our findings also strongly suggest that during pancreas development, the establishment of differentiated acini at the tip of primary branches is a prerequisite for branch bifurcation. Consistently, mutants with impaired acinar differentiation have strong branching defects, with ducts remaining as an interconnected mesh and lacking lateral branches.^38^ Similarly, it would be interesting to investigate if the acquisition of a ductal identity is a prerequisite for cells in a centroacinar position to form cleft and leads to acini split and bifurcation. Interestingly, in Jag1-deficient pancreata, peripheral ductal cells are not properly specified, but secondary branches are still formed.^39^ This suggests that progenitors without a defined ductal identity could still trigger cleft formation or, alternatively, any acinar-proximal cell might take over and induce clefting in Jag1-mutants. Further investigation quantifying acinar fragmentation and duct bifurcations in Jag1-deficient pancreata would be needed to fully assess the requirement of ductal differentiation for clefting and secondary branching.

Additional studies are required to address if such a “protrude and pull” mechanism and a role for tissue fluidity, described here in the pancreas, might extrapolate to other organs. For example, the exocrine pancreas shares similar architecture and secretory function with the salivary gland.^40^^,^^41^ However, despite the overall similarities, morphological differences are evident at the cleft sites.^6^ Salivary glands harbor larger acini with deeper clefts, lined by more cells,^6^ compared with the pancreas. The integrity of the BM is necessary for clefting in both organs, as evidenced by the detrimental effect of collagenase treatments shown in this study for the pancreas and previously reported in the salivary gland.^42^ However, our findings suggest that the reliance on the BM is caused by different underlying morphogenetic mechanisms. We showed here that BM integrity is required in the pancreas for the terminal ductal cells to pull on it to form the clefts. Conversely, it was shown that the salivary gland BM is required to enable the acinar cell layer to adhere, expand, and fold inwardly.^42^ Hence, in the pancreas, clefting is driven by the action of ductal progenitors onto the acinar compartment, while in the salivary gland, clefting is due to an acinar self-organizing mechanism based on preferential cell-matrix adhesion.^42^ Consistently, the perturbation of cell-cell adhesion and/or cell-ECM interactions leads to different cell behaviors in the acinar and ductal compartments of the pancreas and salivary gland.^6^^,^^19^^,^^40^^,^^42^^,^^43^ Nevertheless, none of these experiments selectively targeted either acinar or ductal cellular compartments. Therefore, approaches specifically blocking either acinar cell-matrix interactions in the pancreas or contractility in ductal progenitors in the salivary gland should be employed to assess their respective contribution to clefting in the two glands.

At the molecular level, we identify the IGF/PI3K pathway as a key regulator of cleft-mediated branching in the pancreas. Our results highlighted that the inhibition of IGF/PI3K by LY294002 or linsitinib is linked to increased acinar apoptosis. However, the acinar cell layer integrity does not seem strongly compromised after 24 h of treatment, which is the time when acinar fragmentation by clefting is observed. Also, a similar fragmentation is observed in the *Pdx1*-Cre;*Igf1r*^*flox/flox*^ pancreata in the absence of acinar apoptosis. Hence, apoptosis does not seem to cause an increase in clefting and acinar over-fragmentation but surely contributes to later defects in branching.

Furthermore, we show that when PI3K is overactivated, ductal cells rearrange uncontrollably, which disrupts the epithelial architecture and results in the formation of cysts. The formation of cysts appeared to be linked with acinar-to-ductal transdifferentiation or hybrid acinar-ductal cellular state. This result is in line with the consequences of PI3K overactivation in the adult pancreas that also triggers ductal cyst formation but via the expansion of centroacinar ductal cells instead of transdifferentiation.^44^ This difference in cellular mechanisms could be due to the differences between embryonic and adult tissues. Finally, the IGF signaling is intimately involved in the development and progression of pancreatic cancer.^45^ Whether a similar Igf/Igfr acinar/duct embryonic niche might be exploited in metaplastic events underlying the initiation of pancreatic cancer is an open question, which deserves further investigation.

While primary murine pancreatic ductal adenocarcinoma (PDAC) cells embedded in a collagen matrix have been shown to give rise to organoids that self-organized into highly branched structures,^46^ human pluripotent stem cell (PSC)-derived pancreatic organoids do not fully recapitulate branching morphogenesis as *in vivo*.^47^^,^^48^^,^^49^ Our findings in the mouse underscore the importance of intrinsic epithelial signaling and the cross talk between acinar and terminal-duct cells in these morphogenetic events. Hence, it is conceivable that the limited acinar cell differentiation induced in PSC cultures and/or the lack of a proper acinar/duct niche *in vitro* might hamper cleft formation as well as coordinated bifurcation and elongation of pancreatic branches in organoid models. Our study opens perspectives where fine-tuning of the IGF/PI3K signaling might help recreating the *in vivo* niche, possibly providing a starting point to promote branching in human pancreatic organoids.

### Limitations of the study

This study partly relied on live imaging experiments performed *ex vivo* in mouse embryonic explants cultured on a stiff glass bottom. This experimental setup, although very useful to perform high-quality live imaging, might not be fully representative of the *in vivo* context. In addition, our results suggest that ductal-mediated clefting is more likely to occur in large rather than small acini, and this might be due to the difference in thickness of the surrounding BM. However, further studies are required to experimentally prove this point. Finally, the molecular or cellular mechanisms responsible for positioning the cleft initiation sites in the acini, which appears to be mostly stochastic, remain to be determined.

## STAR★Methods

### Key resources table

**Table:** 

REAGENT or RESOURCE	SOURCE	IDENTIFIER
**Antibodies**		

Rabbit anti-AMYLASE (IHC 1:400)	Merck	Cat #A8273; RRID: AB_258380
Rabbit anti-CASPASE3 (IHC 1:300)	Cell Signaling	Cat #9661; RRID: AB_2341188
Goat anti-COLIV (IHC 1:200)	Merck	Cat #AB769; RRID: AB_92262
Goat anti-CPA1 (IHC 1:500)	Biotechne	Cat #AF2765; RRID: AB_2085841
Rabbit anti-CYTOKERATIN19 (IHC 1:700)	Abcam	Cat #133496;RRID: AB_111552282
Rat anti-E-CADHERIN (IHC 1:600)	Merck	Cat #U3254; RRID: AB_477600
Rabbit anti-GLUCAGON (IHC 1:500)	Immunostar Inc.	Cat #20076; RRID: AB_572241
Goat anti-IGF1 (IHC 1:300)	Biotechne	Cat #AF791; RRID: AB_2248752
Goat anti-IGF1R (IHC 1:200)	Biotechne	Cat #AF305-NA; RRID: AB_354457
Guinea pig anti-INSULIN (IHC 1:2)	Dako	Cat #IR002; RRID: AB_2800361
Rat anti-INTEGRIN α6 (CD49f, VLA-6) (IHC 1:400)	Millipore	Cat #MAB1378; RRID: AB_2128317
Rabbit anti-LAMININ α1 (IHC 1:500)	gift of Prof. T. Sasaki (Oita Uni., Japan)	
Armenian hamster anti-MUCIN (IHC 1:500)	Thermo Scientific	Cat #HM-1630-P1; RRID: AB_54625
Guinea pig anti-NEUROGENIN3 (IHC 1:400)	Merck	Cat #AB10536; RRID: AB_10616222
Mouse anti-NKX6.1 (IHC 1:400)	DSHB	Cat #F55A10; RRID: AB_532378
Goat anti-OSTEOPONTIN (IHC 1:200)	Biotechne	Cat #AF808; RRID: AB_2194992
Rabbit anti-PDGFRβ (Y92) (IHC 1:500)	Abcam	Cat #AB32570; RRID: AB_777165
Rabbit anti-PHOSPHO-AKT (Ser474) (IHC 1:200)	Cell Signaling	Cat #9271; RRID: AB_329825
Rabbit anti-PHOSPHO-HISTONE H3 (Ser10) (IHC 1:300)	Millipore	Cat #06-570; RRID: AB_310177
Rabbit anti-PHOSPHO-MYOSIN (IHC 1:200)	Cell Signaling	Cat #3674; RRID: AB_2147464
Rabbit anti-PTF1A (IHC 1:200)	gift of Prof. C. Wright (Vanderbilt Uni., USA)	
Mouse anti-P120CATENIN (IHC 1:300)	BD Biosciences	Cat #610134; RRID: AB_397537
Rabbit anti-SOX9 (IHC 1:500)	Merck	Cat #AB5535; RRID: AB_2239761
Donkey Alexa Fluor 594-labeled Anti-Goat IgG (IHC 1:750)	Invitrogen	Cat #A11058; RRID: AB_2313737
Goat Alexa Fluor 594-labeled Anti-Guinea Pig IgG (IHC 1:750)	Invitrogen	Cat #A11076; RRID: AB_2534120
Goat DyLight 649-labeled Anti-Armenian Hamster IgG (IHC 1:750)	Dianova	Cat #127-495-099
Donkey Alexa Fluor 488-labeled Anti-Mouse IgG (IHC 1:750)	Invitrogen	Cat #A21202; RRID: AB_141607
Donkey Alexa Fluor 647-labeled Anti-Mouse IgG (IHC 1:750)	Invitrogen	Cat #A31571; RRID: AB_162542
Donkey Alexa Fluor 488-labeled Anti-Rabbit IgG (IHC 1:750)	Invitrogen	Cat #A21206; RRID: AB_2535792
Donkey Alexa Fluor 594-labeled Anti-Rabbit IgG (IHC 1:750)	Invitrogen	Cat #A21207; RRID: AB_141637
Donkey Alexa Fluor 647-labeled Anti-Rabbit IgG (IHC 1:750)	Invitrogen	Cat #A31573; RRID: AB_2536183
Donkey Alexa Fluor 488-labeled Anti-Rat IgG (IHC 1:750)	Invitrogen	Cat #A21208; RRID: AB_2535794

**Biological Samples**		

Gastrointestinal tract dissected from mouse embryos at different stages	This paper	N/A
Pancreatic explants from E12.5 mouse embryos	This paper	N/A

**Chemicals, Peptides, and Recombinant Proteins**		

Hoechst 33342 (250 ng/mL)	Invitrogen	Cat #H1399
LY294002 (20 μM)	Biozol	Cat #ST420-0005
BpV(pic) (2.5 μM)	Sigma	Cat #SML0885
Linsitinib (20-40 μM)	Selleckchem	Cat #S1091
Collagenase type IV (20 μg/mL)	Sigma	Cat #C5138
4-Hydroxytamoxifen (1 μg/mL)	Sigma	Cat #H6278
Alexa Fluor 488-Phalloidin	Invitrogen	Cat #A12379
siR-actin	Spirochrome	Cat #SC001
Rho Kinase inhibitor, Y-27632 (10 μg/ml)	Sigma	Cat #Y0503
N,N,N′,N′-tetrakis(2-hydroxypropyl) ethylenediamine	Sigma	Cat #122262
2,20,20′-nitrilotriethanol	Sigma	Cat #90279

**Deposited Data**		

scRNAseq data	van Gurp et al.^31^	https://scdiscoveries.shinyapps.io/PancDev/ GSE132364

**Experimental Models**		

Mouse: *Krt19-*Cre^ERT^; Krt19tm1(cre/ERT)Ggu	Means et al.^23^	MGI:3797107
Mouse: R26R-mTmG; *B6.129(Cg)-Gt(ROSA)26Sor*^*tm4(ACTB-tdTomato,-EGFP)Luo*^*/J*	Muzumdar et al.^24^	Cat #JAX:037456; RRID:IMSR_JAX:007576
Mouse: Pdx1-Cre; Tg(Pdx1-cre)6Tuv	Hingorani et al.^50^	MGI:3032531
Mouse: Igf1r-Flox; B6;129-Igf1rtm2Arge/J	Dietrich et al.^32^	Cat #JAX:012251; RRID:IMSR_JAX:012251

**Software and Algorithms**		

Fiji ImageJ	NIH	https://imagej.net/software/fiji/
AnalyzeSkeleton	Arganda-Carreras et al.^30^	https://imagej.net/plugins/analyze-skeleton
Imaris version 9.5	BitPlane	https://imaris.oxinst.com/
The R Project for Statistical Computing	CRAN	https://www.R-project.org/
R Studio	R Studio	https://www.rstudio.com/
Zen Digital Imaging for Light Microscopy	Carl Zeiss AG	https://www.zeiss.com/microscopy/en/products/software/zeiss-zen.html

### Resource availability

#### Lead contact

Further information and requests for resources and reagents should be directed to and will be fulfilled by the lead contact, Francesca M. Spagnoli, E-mail: francesca.spagnoli@kcl.ac.uk.

#### Materials availability

This study did not generate new unique reagents.

#### Data and code availability

This study did not report any original code or RNA-sequencing data. Additional information on code used to complete this study is available from the lead contact upon request.

### Experimental models and study participant details

#### Animal work

All procedures relating to animal care and treatment conformed to the Institutional Animal Care and Research Advisory Committee and local authorities (PPL PP6073640, Home Office, UK). All mouse embryos were used without sex identification (mixed sexes). The following mouse strains were used in this study: *Krt19-*Cre^ERT^,^23^ *R26-*mTmG,^24^ *Pdx1-*Cre,^50^ *Igf1r*-flox.^32^ All mice were bred on a C57BL/6J genetic background. Mice were housed in a specific pathogen-free facility in individually ventilated cages. Room temperature was maintained at 22±1°C with 30–70% humidity and lighting followed a 12-h light/dark cycle. Food and water were provided ad libitum and none of the mice had been involved in previous procedures before the study. For timed mating, male and female mice were placed into a breeding cage overnight and plug check was performed daily. The presence of a vaginal plug in the morning was noted as E0.5.

### Method details

#### Immunofluorescence staining

Embryos were collected at E12.5 to E18.5, the abdomen was dissected under a stereomicroscope in cold phosphate buffered saline (PBS) and fixed overnight at 4 °C in 4% paraformaldehyde (PFA). Fixed samples were washed in PBS and cryoprotected overnight in 20% sucrose. Tissues were embedded in OCT compound (Tissue-Tek, Sakura) and sectioned at 12μm thickness. For immunostaining, sections were incubated in TSA (Perkin Elmer) blocking buffer for 1h at room temperature (RT). If necessary, antigen retrieval was performed by boiling slides for 20 min in citrate buffer (Dako). Sections were incubated in primary antibody solution (3% horse serum and 3% BSA in PBS) at the appropriate dilution overnight at 4°C. The following primary antibodies and dilutions were used: Amylase (1:400, Merck A8273), cleaved-Casp3 (1:300, Cell Signaling 9661), Collagen IV (1:200, Merck AB769), Cpa1(1:500, Biotechne AF2765), Cytokeratin19 (1:700, Abcam 133496), E-Cadherin (1:600, Merck U3254), Glucagon (1:500, Immunostar Inc. 20076), Igf1 (1:300, Biotechne AF791), Igf1r (1:200, Biotechne AF305-NA), Insulin (1:2, Dako IR002), Integrin-α6 (1:400, Millipore MAB1378), Laminin-α1 (1:500, kindly provided by Prof. Sasaki, Oita University), Mucin (1:500, Thermo Scientific HM-1630-P1), Neurogenin3 (1:400, Merck AB10536), Osteopontin (1:200, Biotechne AF808), Pdgfrb (1:500, Abcam ab32570) phospho-Akt(Ser473) (1:100, Cell Signaling 9271), phospho-Histone-H3 (1:300, Millipore 06-570), phospho-Myosin (1:200, Cell Signaling 3674), Ptf1a (1:200, kindly provided by Prof. Wright, Vanderbilt University), p120-Catenin (1:300, BD Biosciences 610134), Sox9 (1:500, Merck AB5535).

Sections were washed in PBS and incubated with a combination of Alexa fluor-conjugated secondary antibody (1:750, Invitrogen 11058, A11076, A21202, A21206, A21207, A21208, A31571, A31573; 1:750, Dianova 127-495-099) and counterstained with 250ng/ml of Hoechst 33342, for 1h at RT. When included, Phalloidin (1:400, Invitrogen A12379) was added to the secondary antibody solution. After washes in PBS, sections were mounted with SlowFade Gold Antifade Mountant (ThermoFisher Scientific). To perform multiple rounds of immunofluorescence labeling on the same tissue, antibodies were eluted by treating sections with 1% SDS solution (pH 2) for 1 h at 60°C.^51^ Slides were imaged on a Zeiss LSM 700 confocal microscope using 40×, 63× oil or 10× water immersion objectives.

#### Pancreatic explant culture

Dorsal pancreatic buds were microdissected from mouse embryos at E12.5 and cultured on glass-bottom dishes (MatTek) coated with Fibronectin and filled with Basal Medium Eagle (BME) (10% FBS, 1% Glutamax, 1% Penicillin-Streptomycin, and 50 μg/ml Gentamicin).^21^ Explants were cultured for up to 72h in a tissue incubator (37°C, 5% CO2) and culture medium was changed daily. For explant treatments, BME culture medium was supplemented after 24h with the following small compound molecules: LY294002 (Biozol ST420-0005, final concentration 20 μM), BpV(pic) (Sigma SML0885, final concentration 2.5 μM), linsitinib (Selleckchem S1091, final concentration 20 μM in Figures 4D–4H, S3G, and S3H and 40 μM in Figures 4I and 4J) or Collagenase type IV (Sigma C5138, final concentration 20 μg/ml). Control explants were cultured with BME supplemented with the drug vehicle, DMSO, in the case of LY294002 and linsitinib. Following 24h or 48h of treatment, the explants were briefly washed with PBS, fixed for 20min at 4°C in 4% PFA and either processed for whole-mount immunofluorescence as previously described^21^ or equilibrated overnight in 20% sucrose and embedded in OCT for cryosectioning. Explants were imaged with a Zeiss LSM 700 confocal microscope.

#### Time-lapse image acquisition

Dorsal pancreatic buds were microdissected from *Krt19-*Cre^ERT^;*R26*-mTmG embryos at E12.5 and cultured for 48h in BME supplemented with 4-Hydroxytamoxifen (4-OHT) (Sigma H6278, final concentration 1 μg/ml). Subsequently, 4-OHT was washed out and the explants were cultured in BME with or without the indicated small molecule compound for 1h before starting the acquisition. The microscope environmental chamber set-up was as previously described.^21^ Time-lapse imaging of GFP+ ductal cells was performed overnight for 10-12h on a Zeiss LSM 700 confocal microscope, using a 10X water immersion objective and a viscous immersion fluid (Zeiss Immersol W). 8 μm interval Z-Stacks of 4 to 6 slices were taken every 10min.

Time-lapse imaging of explants treated with Y-27632 (Sigma Y0503, final concentration 10 μg/ml) or non-treated (Figure 2) was performed using an Operetta® CLS™ high content analysis system (PerkinElmer) with a 20× water immersion objective (NA 1.0). 6 μm interval Z-Stacks of 5 slices were taken every 15min during 15h acquisition periods. This set-up enabled the acquisition of time-lapse confocal images on multiple explants in parallel. Explants were cultured in BME supplemented with siR-actin (Spirochrome SC001, 100nM) 24h prior to the acquisition. Subsequently, siR-actin was washed out 1h before starting the time-lapse acquisition and explants were cultured in BME with or without Y-27632 for the duration of the acquisition.

#### Light-sheet microscopy

Whole-mount immunofluorescence labeling of pancreata was performed as previously described.^52^ For Igf1r labeling samples were treated with FLASH2 (80g/L Zwittergent, 250g/L Urea, in 200mM Borate) antigen retrieval solution prior to blocking.^53^ Similar antibodies and dilutions were used as described above.

As described in Glorieux et al.,^52^ CUBIC1 (25% wt/vol urea, 25% wt/vol N,N,N′,N′-tetrakis(2-hydroxypropyl) ethylenediamine, 15% wt/vol Triton X-100, in dH_2_O) and CUBIC2 (50% wt/vol sucrose, 25% wt/vol urea, 10% wt/vol 2,20,20′-nitrilotriethanol, 0.1% vol/vol Triton X-100, in dH_2_O) solutions were used for tissue clarification. Samples were mounted and imaged on a Zeiss Z1 light sheet microscope using 20X acquisition and 10X illumination lenses. 3D renderings were generated with Imaris software (Bitplane Oxford Instruments, version 9.5.1) using the “Crop 3D” tool on the area of interest and the “Surface” module.

#### Image analysis

Structural features and immunostaining intensities were quantified on sections and explants using ImageJ^54^ (rsb.info.nih.gov/ij). The measurement of the spanning angles was manually performed using measurement points in Imaris. The ductal network topologies of explants were analyzed using the “Skeletonize (2D/3D)” and “Analyze Skeleton (2D/3D)” plugins (https://imagej.net/plugins/analyze-skeleton). Prior to the use of plugins 8-bit images of explants were processed as follows. The channel with Opn immunofluorescent labeling was split, a gaussian filter (sigma=2) was applied, the intensity was thresholded (Huang) to create a binary image, and small objects (below 300μm^2^ size) were automatically filtered out. From the data provided by the plugins, we extracted the number of End-points and Triple-points present in ductal networks and normalized them by the total number of branches. The End-points and Triple-points represent a good approximation of the number of terminal branches and trifurcations, respectively, in the ductal networks.

The tracking of clefts over a 48h time-course in *Pdx1-*Cre;*R26*mTmG pancreatic explants was performed manually with ImageJ. ROIs delineating the pancreatic epithelium edges were drawn, aligned, and centered at t= 0, t= 24h and t=48h. Clefts detected at t=0h were tracked at t=24h and t=48h to analyze if they formed bifurcated branches or not. The ROIs were used to quantify the growth of pancreatic explants after 48h of treatment with LY294002 or linsitinib in comparison to DMSO treatment (drug vehicle) alone.

The membrane intensity of Phalloidin, E-Cadherin and phospho-Myosin was quantified by measuring the mean pixel intensity in manually drawn regions of interest (ROI) delineating ductal membranes. The intensity of phospho-AKT was quantified by measuring the mean pixel intensity in manually drawn ROIs delineating the pancreatic epithelium. These values were normalized by the mean Hoechst intensity of cells quantified and averaged per explant.

*Krt19*-mGFP^+^ ductal cells were tracked in explants using Imaris. To quantify ductal cell displacement their surfaces were automatically generated using the “Surface” module on all focal planes (z-stacks of 4 to 6 slices with 8μm interval). Filters were applied to discard multicellular objects and unspecific fluorescent speckles (“Number of voxels” greater than 100 and “Area” comprised between 500 μm^2^ and 4300 μm^2^). Objects which were detected for less than 80 min or were detected discontinuously (gaps of more than 50 min present in their tracks) were filtered out. Any explant drift was corrected using the “Correct drift” tool on object tracks, which also corrected the image drift. The displacement of cells was measured using the “Detailed Statistics” tool and extracted as a .csv file. For generating Video S7, we used the “Spots creation” module and manually edited the tracks.

#### AFM measurements

AFM measurements were carried out using a Bioscope atomic force microscope (BioScope Resolve BioAFM, Bruker), coupled with an inverted fluorescence microscope (Nikon Eclipse Ti-U). Fresh mouse *Pdx1-Cre*;mTmG Tg pancreatic explants were adhered to glass bottom WillCo-dishes (Willco Wells, GWST-5040). Before measuring, the explants were washed with PBS three times. Seven explants were measured, selecting in each case at least three acinar and cleft sites. A spherical nitride tip (5 μm) on a nitride pre-calibrated cantilever was used. For each experiment, the deflection sensitivity was calibrated. For each measurement, an area of 5 μm x 5 μm was selected, and 8x8 force-extension measurements were conducted to probe the stiffness of the region. Each measurement consisted of a 7.5 μm ramp up to a maximum trigger force of 10 nN, approached and retracted at a velocity of 10 μm s^-1^. The Young’s modulus *E* for each probed region was calculated by fitting the force-extension curves to a Hertz model with spherical geometry, as:

$$F=\frac{4ER^{1/2}}{3\left(1-\nu^{2}\right)}\delta^{3/2}$$

where *R* is the radius of the tip, *δ* the indentation depth, and *ν* = 0.5 the Poisson’s ratio. Only the region between 20% and 80% of the maximum force was employed for fitting. Curves showing abnormal or artifactual patterns were discarded. Typically, ∼50 Young modulus measurements were used per area.

#### scRNAseq dataset analysis

The scRNA-seq data has been previously published in van Gurp et al.^31^ Uniform Manifold Approximation and Projection (UMAP) visualizations were imported from https://scdiscoveries.shinyapps.io/PancDev/. The Seurat object was downloaded from GSE132364.^31^ Violin plots were generated using the Seurat package splitting cells by embryonic stages.

### Quantification and statistical analysis

All statistical analyses were done using the R package rstatix, and statistical details related to tests performed and sample size are specified in the figure legends. For box-plots, the elements shown are the 25% (Q1, upper box boundary), 50% (median, black line within the boxes) and 75% (Q3, lower box boundary) quartiles, and the whiskers represent a maximum of 1.5X the interquartile range. p-values were calculated using two-tailed t-tests. Normality was assessed using the Shapiro-Wilk normality test. Statistical significance was calculated using unpaired Student’s *t*-tests for data displaying normal distributions and Mann-Whitney *U*-tests otherwise. In all experiments involving drug perturbations, experiments were repeated on explants collected from at least two embryonic litters.

## Supplemental information

Document S1. Figures S1–S5

Document S2. Article plus supplemental information
