# Supplementary material for: Acinar-ductal cell rearrangement drives branching morphogenesis of the murine pancreas in an IGF/PI3K-dependent manner
Source: Dev Cell. 2024 Feb 5;59(3):326–338.e5. doi: 10.1016/j.devcel.2023.12.011 (PMC11805742; doi:10.1016/j.devcel.2023.12.011)
Supplement: Document S1. Figures S1–S5 [file mmc1.pdf]

Developmental Cell, Volume *59*

Supplemental information

Acinar-ductal cell rearrangement drives branching morphogenesis of the murine pancreas in an IGF/PI3K-dependent manner

Jean-Francois Darrigrand, Anna Salowka, Alejo Torres-Cano, Rafael Tapia-Rojo, Tong Zhu, Sergi Garcia-Manyes, and Francesca M. Spagnoli

SUPPLEMENTAL INFORMATION

Acinar-ductal cell rearrangement drives branching morphogenesis of the murine pancreas in an IGF/PI3K-dependent manner

Jean-Francois Darrigrand[1], Anna Salowka[1], Alejo Torres Cano[1], Rafael Tapia-Rojo[2], Tong Zhu[3,4], Sergi Garcia-Manyes[3,4], Francesca M. Spagnoli[1,5*]

[1]Centre for Gene Therapy and Regenerative Medicine, King's College London, London, Great Maze Pond, SE1 9RT, London, UK; [2]Department of Physics, London Centre for Nanotechnology, King's College London, London, UK; [3]Department of Physics, Randall Centre for Cell and Molecular Biophysics, Centre for the Physical Science of Life and London Centre for Nanotechnology, King's College London, London, UK; [4]Single-Molecule Mechanobiology Laboratory, The Francis Crick Institute, London, UK.

[5]Lead contact

*Correspondence: francesca.spagnoli@kcl.ac.uk

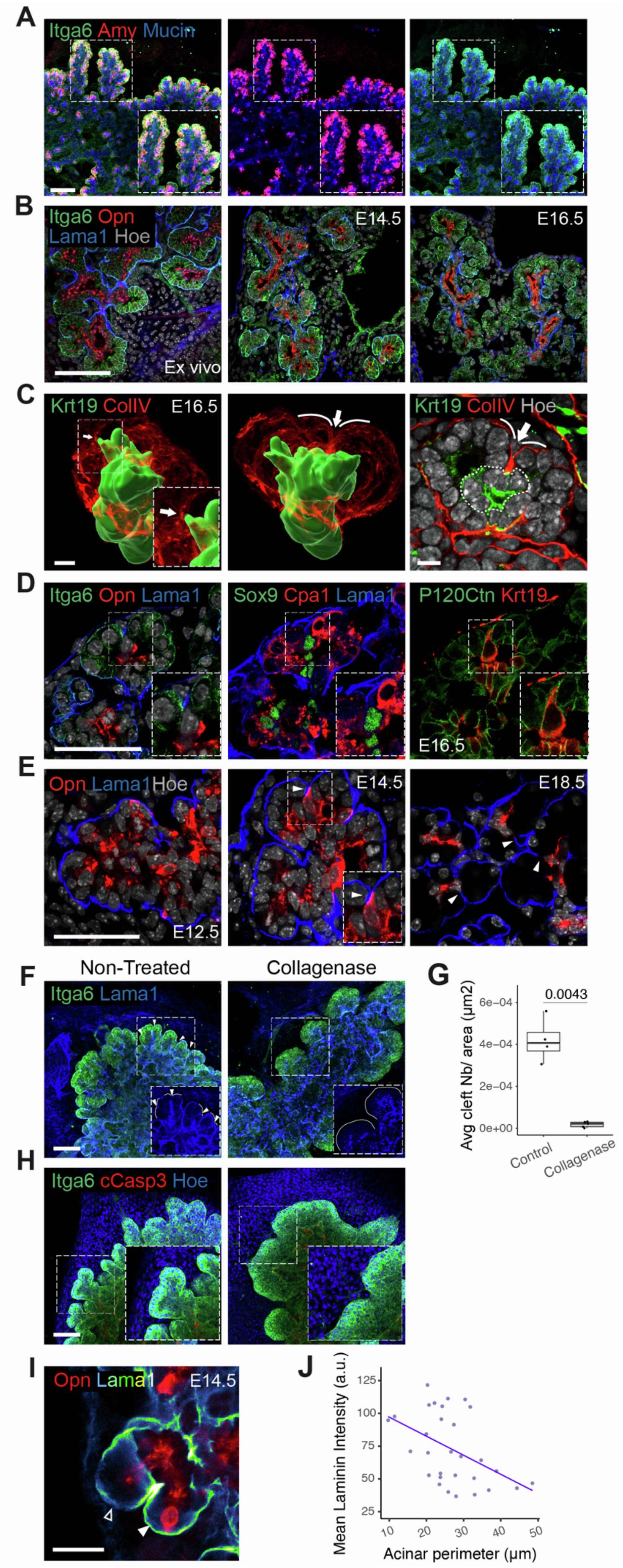
A
Itga6 Amy Mucin
B
Itga6 Opn
Lama1 Hoe
Ex vivo
E14.5
E16.5
C
Krt19 ColIV
E16.5
Krt19 ColIV Hoe
D
Itga6 Opn Lama1
Sox9 Cpa1 Lama1
P120Ctn Krt19
E16.5
E
Opn Lama1 Hoe
E12.5
E14.5
E18.5
F
Non-Treated
Collagenase
Itga6 Lama1
G
0.0043
Avg cleft Nb/ area (µm2)
6e−04
4e−04
2e−04
0e+00
Control
Collagenase
H
Itga6 cCasp3 Hoe
I
Opn Lama1
E14.5
J
Mean Laminin Intensity (a.u.)
125
100
75
50
25
10
20
30
40
50
Acinar perimeter (µm)

Figure S1. Characterization of pancreatic clefts and branches during embryonic development, related to Figure 1.

(A) Representative confocal images of an *ex vivo* pancreatic explant collected at E12.5, cultured for 48h and immunostained for Integrin alpha6 (Itga6), Amylase (Amy) and Mucin. Insets show higher magnifications of boxed regions. Different channel combinations are shown highlighting the colocalization between Itga6 and Amy in acinar cells. Scale bar, 100μm.

(B) Representative confocal images of an *ex vivo* cultured pancreatic explant (left panel) and embryonic pancreatic tissue at E14.5 (middle panel) and E16.5 (right panel). All samples were immunostained for Itga6, Osteopontin (Opn) and Laminin alpha-1 (Lama1). Hoechst (Hoe) was used as nuclear counterstain. *Ex vivo* cultures recapitulate the *in vivo* pancreatic tissue architecture, as previously reported[21,22]. Scale bar, 100μm.

(C) Representative light-sheet images of E16.5 pancreatic tissue immunostained for Cytokeratin 19 (Krt19) and Collagen IV (ColIV). Left, Middle: 3D volumetric rendering showing Krt19$^+$ ductal cells (green) and basement membrane (BM) (red). Arrows point at BM lining clefts in direct contact with a ductal cell below. Scale bar, 50μm. Right panel: focal plane of the light-sheet Z-stack showing the 2D architecture of cleft. N=3 wholemount pancreata were analyzed. Scale bar, 10μm.

(D) Representative confocal images of multiplex immunostaining on E16.5 pancreatic tissue. The same tissue section underwent sequential immunostaining with different antibody combinations: Itga6, Opn and Lama1 (left panel), Cpa1, Sox9 and Lama1 (middle panel), p120Ctn and Krt19 (right panel). Insets show higher magnifications of the boxed region highlighting a protruding ductal cell positive for canonical ductal markers (Opn, Sox9, Krt19) positioned below a cleft. Hoechst was used as nuclear counterstain and shown in grey. Scale bar, 50μm. N=sections from 3 pancreata were analyzed.

(E) Representative confocal images of E12.5, E14.5 and E18.5 pancreatic tissue, immunostained for Opn and Lama1. Clefts (arrowheads) are visible at E14.5 and E18.5. Sections from 3 pancreata were analyzed per developmental stage. Scale bar, 50μm.

(F) Representative confocal images of pancreatic explants non-treated or treated with Collagenase type IV and immunostained for Itga6 and Lama1. Arrowheads indicate cleft sites. Insets show higher magnifications of the boxed regions. Scale bar, 100μm.
(G) Quantification of clefts in non-treated control pancreatic explants or upon Collagenase type IV-treatment. Average clefts number (Nb) is shown as relative to the total number of acini analyzed. N= 4-5 explants per treatment. Student's *t*-tests.
(H) Representative confocal images of pancreatic explants treated with Collagenase type IV or non-treated controls and immunostained for Itga6 and cCasp3. Insets show higher magnifications of the boxed regions. Scale bar, 100μm. N= 4-5 explants per treatment.
(I) Representative confocal image of E14.5 pancreatic tissue immunostained for Opn and Lama1. Lama1 staining is pseudo-colored with the "Green Fire" LUT. Open arrowhead indicates large acinus; filled arrowheads indicates small acinus. N=3 wholemount pancreata were analyzed. Scale bar, 10μm.
(J) Quantification of the mean Lama1 fluorescence intensity along the basal perimeter of acini plotted against the acini perimeter. Laminin fluorescence signal intensity was used as a proxy for BM thickness. Measurements were performed on 3 wholemount E14.5 pancreata. Linear regression suggests that Lama1 intensity decreases as acini perimeter increases. Hence, very big acini having a thinner BM might be more prone to BM pulling by ductal cells, which would intrinsically control the splitting of acini when they become too big.

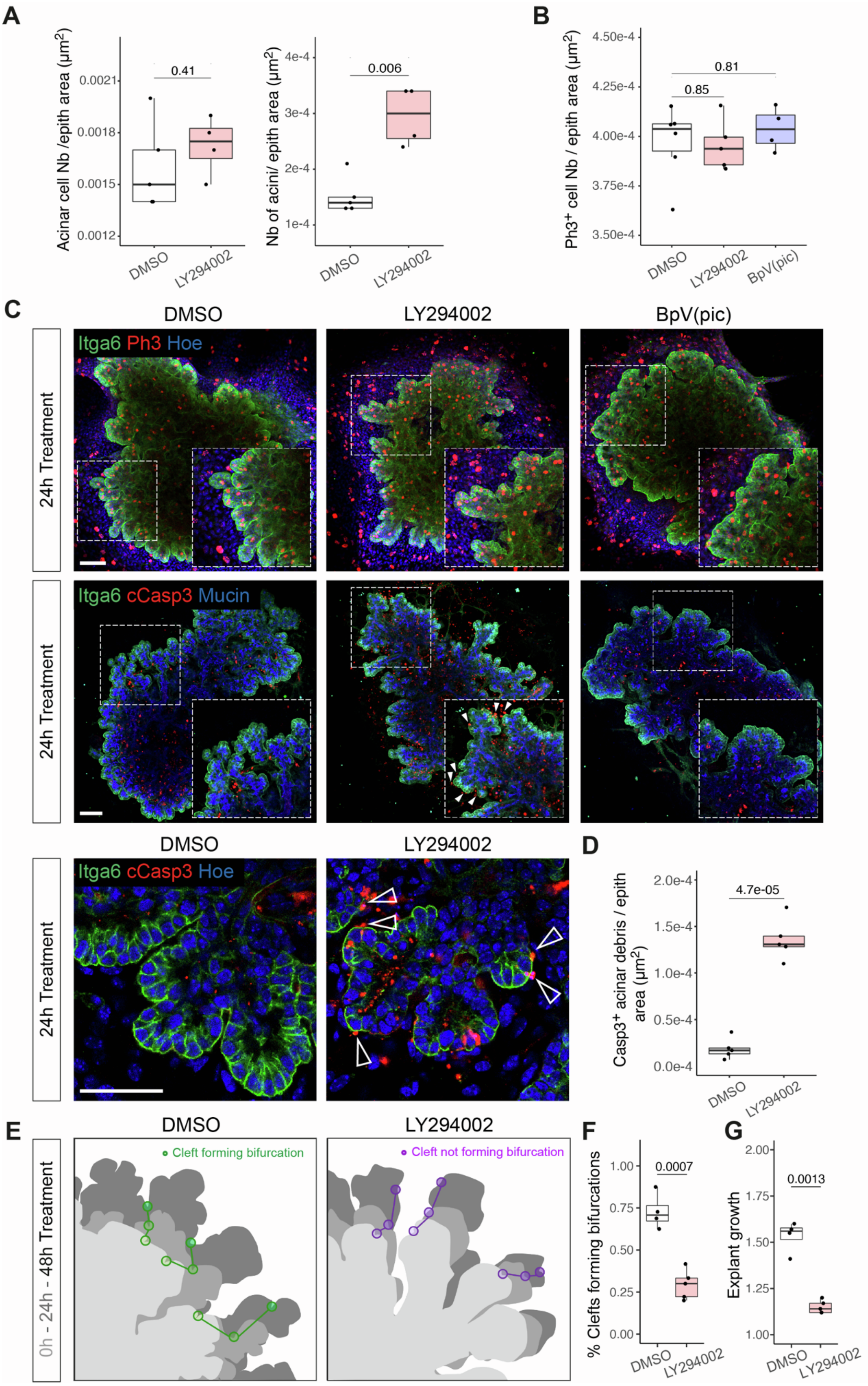

Figure S2. Impact of PI3K dysregulation on cell proliferation and apoptosis in pancreatic explants, related to Figure 3.

(A) Quantification of number of acinar cells (left) and acini (right) in pancreatic explants treated with DMSO (Control) or LY294002 for 24h. Numbers are expressed relative to the epithelial area of the explants (in μm^2). N= 4-5 explants per treatment. Student's *t*-tests.

(B) Quantification of Phospho-histone H3 $(Ph3)^+$ cells in pancreatic explants treated with DMSO (Control), LY294002 or BpV(pic) for 24h. Numbers are expressed relative to the epithelial area of the explants (in μm^2). N= 4-6 explants per treatment. Student's *t*-tests.

(C) Representative confocal images of pancreatic explants collected at E12.5, exposed for 24h to indicated treatments and immunostained for Itga6 and Ph3 (top) or Itga6 and cCasp3 with (middle) or without (bottom) Mucin. Insets show higher magnifications of boxed regions. $cCasp3^+$ apoptotic debris (arrowheads) is detected next to the acini in LY294002-treated explants. Scale bar, 100μm. Bottom panel: magnified images of pancreatic explants upon indicated treatments. Arrowheads indicate $cCasp3^+$ debris extruded from acinar structures. Scale bar, 50μm.

(D) Quantification of $cCasp3^+$ apoptotic debris in contact with $Itga6^+$ acinar cells in pancreatic explants upon indicated treatments. Values are expressed relative to the epithelial area of the explants (in μm^2). N= 5 explants per treatment. Student's *t*-test.

(E) Representative time-lapse binary masks of *Pdx1*-Cre;*R26*mTmG pancreatic explants cultured for 48h with DMSO (Control) or LY294002 and imaged every 24h. The time of imaging of the explants is color-coded in grayscale. Clefts detected in the explants at t=0h were tracked at later time-points. Clefts, which formed a bifurcation by the end of imaging (t= 48h), are marked with a green circle, or with a violet circle when they did not. Green and violet lines join the location points of clefts at each time point.

(F) Quantification of clefts forming bifurcations during the 48h time-course relative to the total number of clefts detected at t= 0h in pancreatic explants cultured with DMSO (Control) or LY294002.

(G) Ratio of the size of pancreatic explants measured after 48h of culture with DMSO (Control) or LY294002 compared to their size after 24h of culture. N=4-5 explants per treatment. Student's *t*-tests.

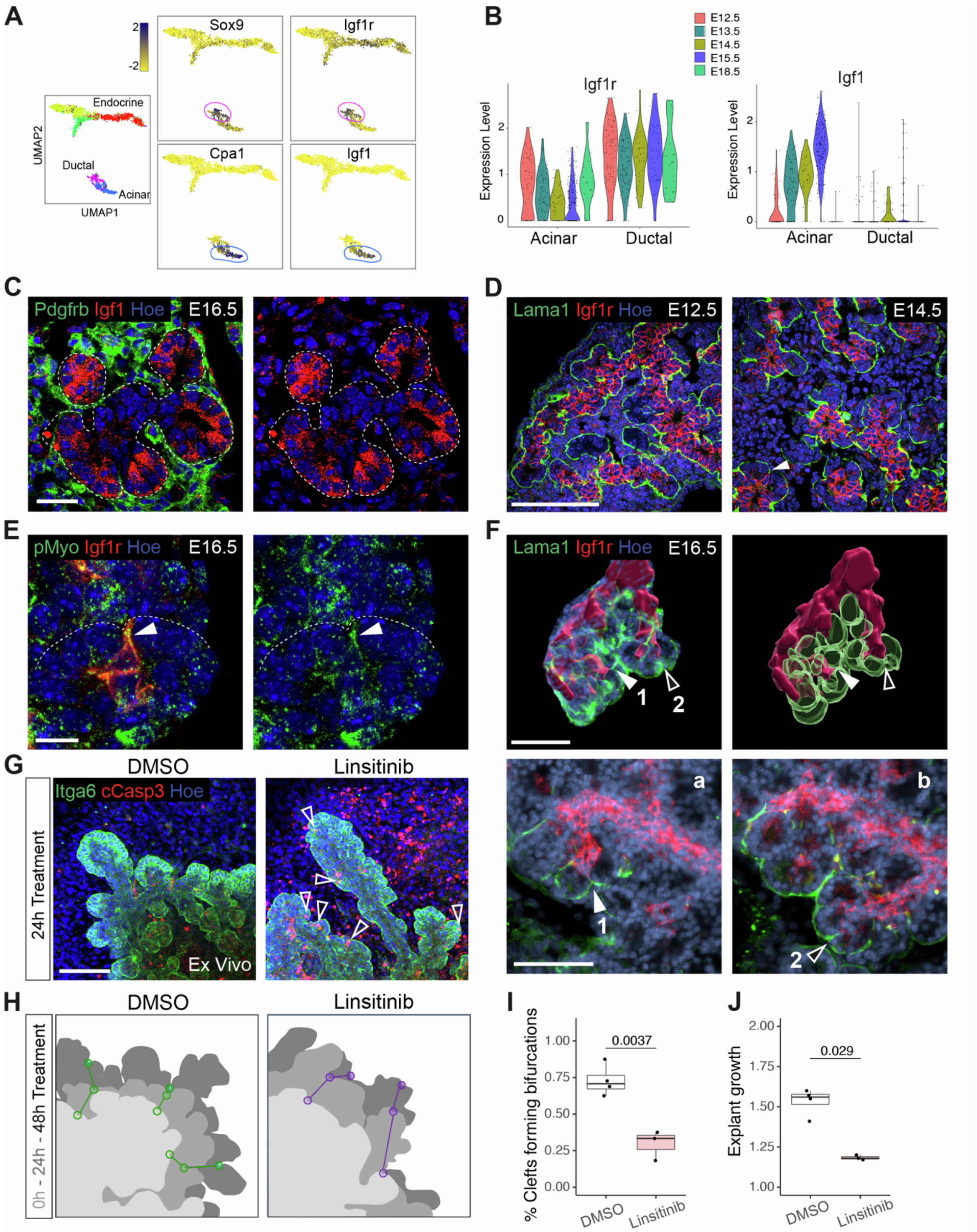

Figure S3. Igf1r specifically marks ductal cells in the developing pancreas, related to Figure 4.

(A) Left panel: Uniform Manifold Approximation and Projection (UMAP) visualization representing pancreatic epithelial cells isolated from mouse embryos between stages E12.5 to E18.5 and color-coded based on their identity[31]. Right panels: Expression of genes marking the ductal (*Sox9* and *Igf1r*) and acinar (*Cpa1* and *Igf1*) clusters.

(B) Distribution of expression of *Ig1r* and *Igf1* in cells from the acinar and ductal clusters at E12.5, E13.5, E14.5, E15.5 and E18.5. The violin plots are color-coded according to the cell developmental stages. Expression on the *y*-axis represents log-normalized expression values.

(C) Representative confocal image of E16.5 pancreatic tissue immunostained for Igf1 and Pdgfrb. The white dotted line delineates the border between the pancreatic epithelium and surrounding mesenchyme. Scale bar, 25μm.

(D) Representative confocal images of E12.5 and E14.5 pancreatic tissue immunostained for Igf1r and Lama1. A cleft (arrowhead) is visible in the E14.5 tissue. Scale bar, 100μm.

(E) Representative confocal image of E16.5 pancreatic tissue immunostained for pMyo and Igf1r. The white line delineates the basal side of an acinus; arrowhead indicates p-Myo accumulation in an Igf1r+ ductal cell protrusion. Scale bar, 10μm.

(F) Representative light-sheet images of E16.5 pancreatic tissue immunostained for Lama1 and Igf1r. Top panels: 3D rendering showing $Igf1r^+$ ductal structures (red isosurface) and $Lama1^+$ BM surrounding the acinar structures (green isosurface). Arrowheads 1 and 2 point at clefts localized at the distal end of terminal branches. Bottom panels: focal planes of the light-sheet Z-stack showing the 2D architecture of cleft 1 (a) and cleft 2 (b). Scale bar, 100μm.

(G) Representative confocal images of pancreatic explants collected at E12.5, cultured for 24h with DMSO (Control) or Linsitinib and immunostained for Itga6 and cCasp3. $cCasp3^+$ apoptotic debris (arrowheads) is detected in the acini of Linsitinib-treated explants. Scale bar, 100μm.

(H) Representative time-lapse binary masks of *Pdx1*-Cre;*R26*mTmG pancreatic explants cultured for 48h with DMSO (Control) or Linsitinib and imaged every 24h. The time of imaging of the explants is color-coded in grayscale. Clefts detected in the explants at t=0h were tracked at later time-points. Clefts, which formed a bifurcation by the end of imaging (t= 48h), are marked with a green circle, or with a violet circle when they did not. Green and violet lines join the location points of clefts at each time point.

(I) Quantification of clefts forming bifurcations during the 48h time-course relative to the total number of clefts detected at t= 0h in pancreatic explants cultured with DMSO (Control) or Linsitinib.

(J) Ratio of the size of pancreatic explants measured after 48h of culture with DMSO (Control) or Linsitinib compared to their size measured after 24h of culture. N= 3-4 explants per treatment. Student's *t*-tests.

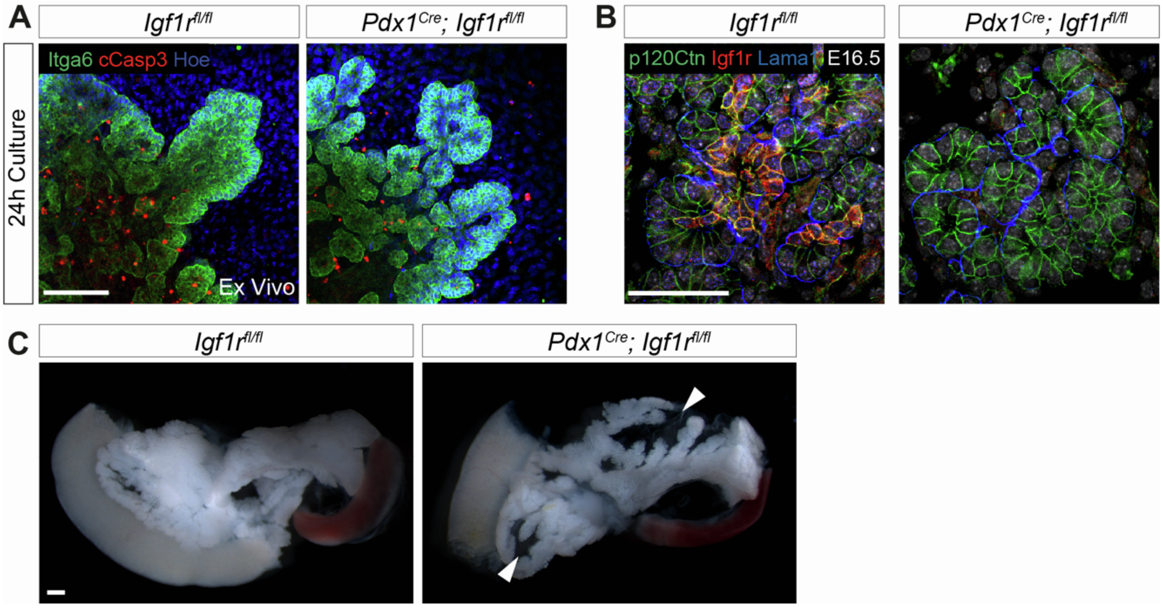

Figure S4. Characterization of *Pdx1*-Cre;*Igf1r*$^{flox/flox}$ pancreatic tissue, related to Figure 5.

(A) Representative confocal images of *Igf1r*$^{flox/flox}$ and *Pdx1*-Cre;*Igf1r*$^{flox/flox}$ pancreatic explants immunostained for Itga6 and cCasp3. Scale bar, 100μm.

(B) Representative confocal images of *Igf1r*$^{flox/flox}$ and *Pdx1*-Cre; *Igf1r*$^{flox/flox}$ pancreatic sections at E16.5 immunostained for p120Ctn, Igf1r and Lama1. *Pdx1-Cre;Igf1r*$^{flox/flox}$ mice showed a loss of *Igf1r* expression in the vast majority of pancreatic ductal cells. Scale bar, 50μm.

(C) Representative stereomicroscope images of *Igf1r*$^{flox/flox}$ and *Pdx1*-Cre;*Igf1r*$^{flox/flox}$ pancreata at E18.5. Arrowheads indicate defects in long lateral branches in the mutant pancreas. Scale bar, 1mm.

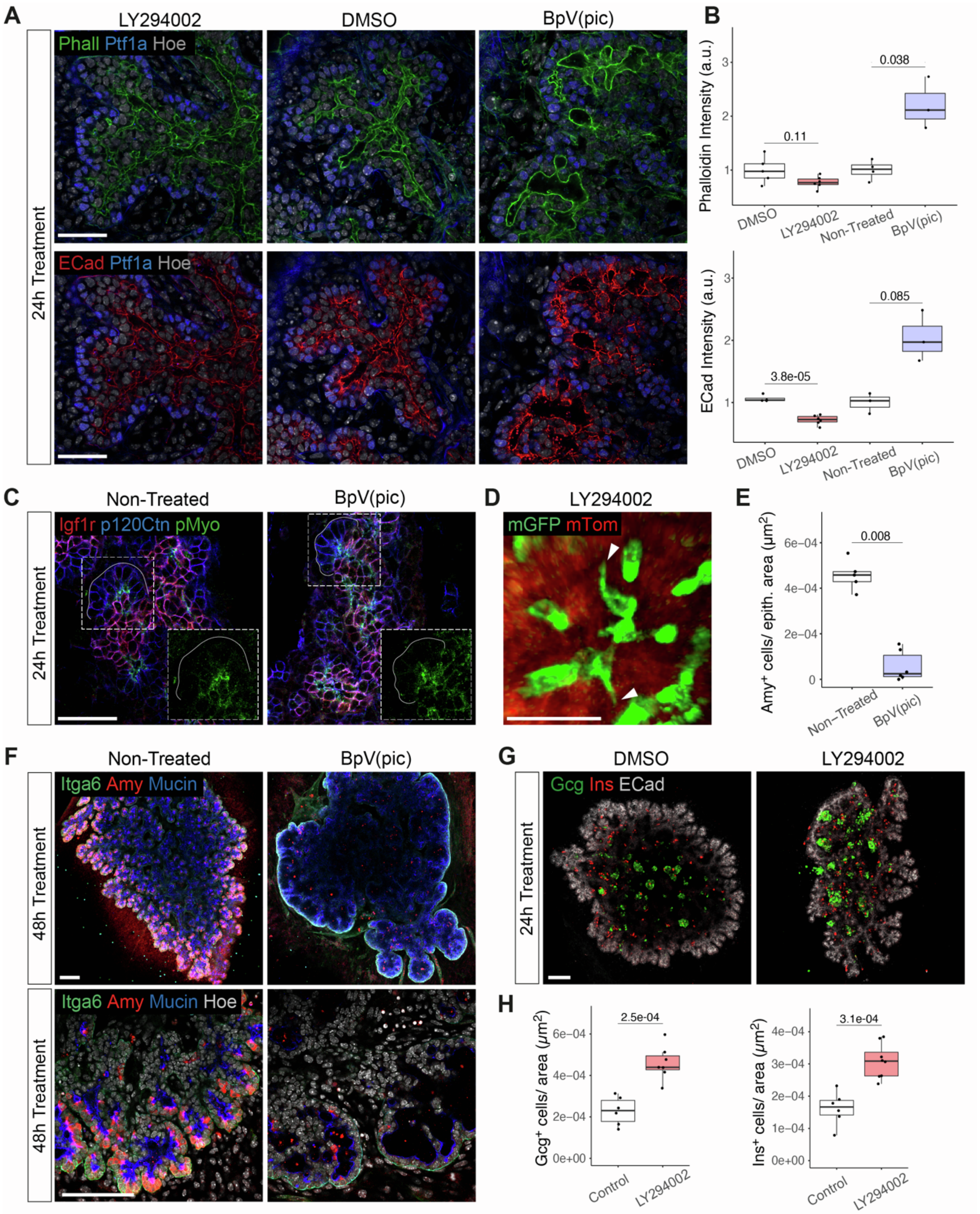

Figure S5. PI3K dysregulation affects actomyosin dynamics in ductal cells and pancreatic cell fate acquisition, related to Figure 6.

(A) Representative confocal images of cryosections of pancreatic explants treated for 24h with DMSO (Control), LY294002 or BpV(pic) and immunostained for either Phalloidin (Phall) (top panel) or E-Cadherin (Ecad) (bottom panel) and Ptf1a. Scale bar, 50μm.

(B) Quantification of Phalloidin (top panel) and E-Cadherin (bottom panel) fluorescence intensity at the apical membrane of ductal cells in pancreatic explants upon indicated treatments. In this tissue-context, an increase in ductal fluidity did not coincide with a decrease in ductal cell adhesion. N= 3-6 explants per treatment. Student's *t*-tests.

(C) Representative confocal images of cryosections of pancreatic explants treated for 24h with BpV(pic) or non-treated (control) and immunostained for Igf1r, p120Ctn and pMyo. Insets show higher magnifications of the boxed regions, displaying pMyo separated channel (green). Continuous white line delineates the basal side of an acinus. Scale bar, 50μm.

(D) Representative confocal time-lapse image of *Krt19*-Cre^{ERT};*R26*mTmG pancreatic explants treated for 24h with LY294002. Arrowheads indicate $mGFP^+$ ductal cells (green) retaining the ability to form protrusions upon LY294002 exposure. Scale bar, 20μm.

(E) Quantification of Amy^+ cells in pancreatic explants upon indicated treatments. Numbers are expressed relative to the epithelial area of explants (in μm^2). N= 5-6 explants per treatment. Mann-Whitney *U*-test.

(F) Representative confocal images of pancreatic explants treated for 48h with BpV(pic) or non-treated (control) and immunostained for Itga6, Amy and Mucin. Bottom panel shows higher magnification of explants. Scale bar, 100μm.

(G) Representative confocal images of pancreatic explants treated for 24h with DMSO (Control) or LY294002 and immunostained for Glucagon (Gcg), Insulin (Ins) and ECad. Scale bar, 100μm.

(H) Quantification of Gcg^+ (left) and Ins^+ (right) cells in pancreatic explants upon indicated treatments. Numbers are expressed relative to the epithelial area of explants (in μm^2). N= 6-8 explants per treatment. Student's *t*-tests.
